# Physical inactivity, cardiometabolic disease, and risk of dementia: an individual-participant meta-analysis

**DOI:** 10.1136/bmj.l1495

**Published:** 2019-04-17

**Authors:** Archana Singh-Manoux, Jaana Pentti, Séverine Sabia, Solja T Nyberg, Lars Alfredsson, Marcel Goldberg, Anders Knutsson, Markku Koskenvuo, Aki Koskinen, Anne Kouvonen, Maria Nordin, Tuula Oksanen, Timo Strandberg, Sakari B Suominen, Töres Theorell, Jussi Vahtera, Ari Väänänen, Marianna Virtanen, Peter Westerholm, Hugo Westerlund, Marie Zins, Sudha Seshadri, G David Batty, Pyry N Sipilä, Martin J Shipley, Joni V Lindbohm, Jane E Ferrie, Markus Jokela, Mika Kivimäki

**Affiliations:** 1 Department of Epidemiology and Public Health, University College London, London WC1E 6BT, UK; 2 Clinicum, Faculty of Medicine, FI-00014 University of Helsinki, Helsinki, Finland; 3 Helsinki Institute of Life Science, University of Helsinki, Helsinki, Finland; 4 INSERM U1153, Epidemiology of Ageing and Neurodegenerative diseases, Paris, France; 5 Department of Public Health, University of Turku, Turku, Finland; 6 Institute of Environmental Medicine, Karolinska Institutet, Stockholm, Sweden; 7 Inserm UMS 011, Population-Based Epidemiological Cohorts Unit, Villejuif, France; 8 Department of Health Sciences, Mid-Sweden University, Sundsvall, Sweden; 9 Finnish Institute of Occupational Health, Helsinki, Finland; 10 Faculty of Social Sciences, University of Helsinki, Helsinki, Finland; 11 SWPS University of Social Sciences and Humanities in Wroclaw, Wroclaw, Poland; 12 Administrative Data Research Centre (Northern Ireland), Centre for Public Health, Queen's University Belfast, Belfast, UK; 13 Stress Research Institute, Stockholm University, Stockholm, Sweden; 14 Department of Psychology, Umeå University, Umeå, Sweden; 15 Helsinki University Hospital, Helsinki, Finland; 16 Center for Life Course Health Research, University of Oulu, Oulu, Finland; 17 University of Skövde, Skövde, Sweden; 18 Turku University Hospital, Turku, Finland; 19 School of Educational Sciences and Psychology, University of Eastern Finland, Joensuu, Finland; 20 Department of Medical Sciences, Uppsala University, Uppsala, Sweden; 21 Glenn Biggs Institute for Alzheimer’s and Neurodegenerative Diseases, University of Texas Health Sciences Center, San Antonio, TX, USA; 22 Framingham Heart Study, Framingham, MA, USA; 23 Bristol Medical School, Population Health Sciences, University of Bristol, UK; 24 Biomedicum, Faculty of Medicine, University of Helsinki, Helsinki, Finland

## Abstract

**Objective:**

To examine whether physical inactivity is a risk factor for dementia, with attention to the role of cardiometabolic disease in this association and reverse causation bias that arises from changes in physical activity in the preclinical (prodromal) phase of dementia.

**Design:**

Meta-analysis of 19 prospective observational cohort studies.

**Data sources:**

The Individual-Participant-Data Meta-analysis in Working Populations Consortium, the Inter-University Consortium for Political and Social Research, and the UK Data Service, including a total of 19 of a potential 9741 studies.

**Review method:**

The search strategy was designed to retrieve individual-participant data from prospective cohort studies. Exposure was physical inactivity; primary outcomes were incident all-cause dementia and Alzheimer’s disease; and the secondary outcome was incident cardiometabolic disease (that is, diabetes, coronary heart disease, and stroke). Summary estimates were obtained using random effects meta-analysis.

**Results:**

Study population included 404 840 people (mean age 45.5 years, 57.7% women) who were initially free of dementia, had a measurement of physical inactivity at study entry, and were linked to electronic health records. In 6.0 million person-years at risk, we recorded 2044 incident cases of all-cause dementia. In studies with data on dementia subtype, the number of incident cases of Alzheimer’s disease was 1602 in 5.2 million person-years. When measured <10 years before dementia diagnosis (that is, the preclinical stage of dementia), physical inactivity was associated with increased incidence of all-cause dementia (hazard ratio 1.40, 95% confidence interval 1.23 to 1.71) and Alzheimer’s disease (1.36, 1.12 to 1.65). When reverse causation was minimised by assessing physical activity ≥10 years before dementia onset, no difference in dementia risk between physically active and inactive participants was observed (hazard ratios 1.01 (0.89 to 1.14) and 0.96 (0.85 to 1.08) for the two outcomes). Physical inactivity was consistently associated with increased risk of incident diabetes (hazard ratio 1.42, 1.25 to 1.61), coronary heart disease (1.24, 1.13 to 1.36), and stroke (1.16, 1.05 to 1.27). Among people in whom cardiometabolic disease preceded dementia, physical inactivity was non-significantly associated with dementia (hazard ratio for physical activity assessed >10 before dementia onset 1.30, 0.79 to 2.14).

**Conclusions:**

In analyses that addressed bias due to reverse causation, physical inactivity was not associated with all-cause dementia or Alzheimer’s disease, although an indication of excess dementia risk was observed in a subgroup of physically inactive individuals who developed cardiometabolic disease.

**Figure:**
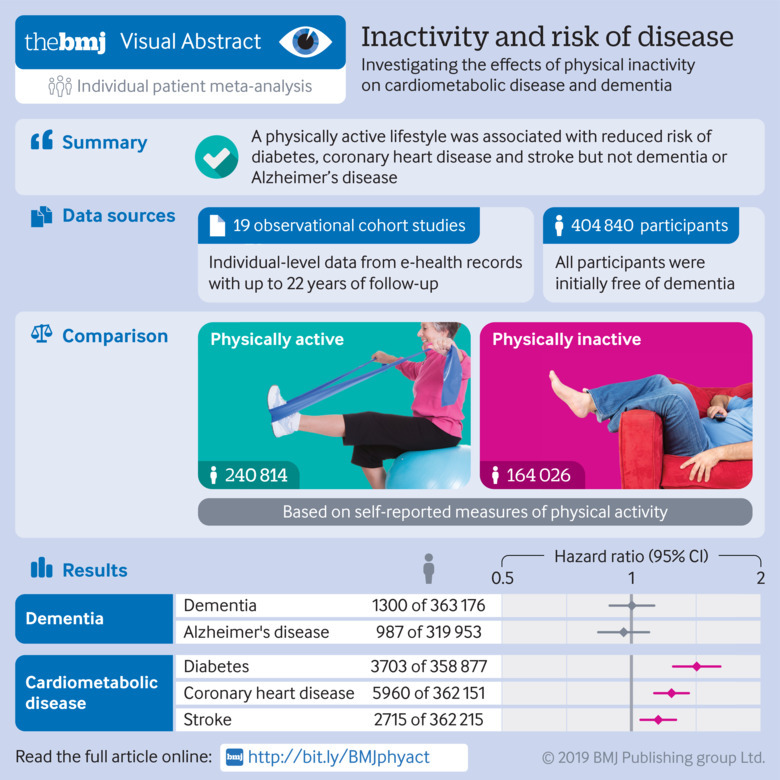


## Introduction

Evidence that physical inactivity is a risk factor for dementia remains uncertain. Randomised controlled trials have linked physical activity to better cognitive performance, but they have not shown reduced risk of dementia or Alzheimer’s disease.^1^ Observational studies suggest an approximately 1.2-fold increased risk of diabetes and major cardiovascular disease in physically inactive individuals. Those who develop these diseases, in turn, have an increased risk of dementia (the summary hazard ratio varies between 1.3 and 2.2 for diabetes,^2^ and is 1.3 for coronary heart disease^3^ and 2.2 for stroke^4^). On the basis of this indirect evidence, the expected hazard ratio for the association of physical inactivity with dementia would be negligible, varying between 1.05 and 1.14 (see appendix, page 2).

Studies that have examined the direct association between physical inactivity and dementia report much higher effect estimates, the summary hazard ratio being 1.3 to 1.5 for physically inactive individuals.^5^ ^6^ ^7^ These estimates may be inflated by reverse causation bias, because most studies had a follow-up duration of less than 10 years.^8^ ^9^ ^10^ This means that physical inactivity assessment was undertaken during the preclinical or prodromal stage of dementia, which is characterised by a decline in physical activity.^11^ ^12^

To address these uncertainties, we investigated the association between physical inactivity and dementia using individual-level data from 19 cohort studies with long follow-up for morbidity and mortality. A standard method to reduce reverse causation is to exclude outcomes occurring in the initial follow-up period. Thus, we determined the association between physical inactivity and dementia for a population with physical activity measured a minimum of 10 years before dementia onset. In addition, we also examined associations between physical inactivity, incident cardiometabolic disease, and subsequent dementia to elucidate this plausible trajectory of risk.

## Methods

### Cohort selection and data extraction

We conducted an individual-participant meta-analysis according to PRISMA guidelines. The 19 prospective cohort studies for which relevant data on physical inactivity and dementia were available were identified using an electronic search of the Individual-Participant-Data Meta-analysis in Working Populations (IPD-Work) Consortium,^13^ the Inter-University Consortium for Political and Social Research (www.icpsr.umich.edu/icpsrweb/ICPSR/) and the UK Data Service (http://ukdataservice.ac.uk/) (16 January 2018). Exposure search terms were “physical activity” and “exercise” and outcome search terms “dementia,” “Alzheimer’s disease,” and “mortality.” For additional individual-level data, we contacted principal investigators of the IPD-Work consortium.

Inclusion criteria for cohort studies were: prospective cohort study with assessment of physical inactivity at baseline; >10 year follow-up for all-cause dementia or Alzheimer’s disease; and incident dementia cases recorded both during the first 10 years of follow-up and, among those free of dementia at that point, incident cases of dementia during follow-up starting from year 10.

Individual-level data extracted included physical activity, sociodemographic characteristics, lifestyle factors, prevalent dementia and cardiometabolic disease at baseline, and incident dementia, cardiometabolic disease, and death at follow-up.

### Assessment of physical inactivity and baseline covariates

Leisure-time physical activity at baseline was self reported.^14^ Some studies had general questions about time spent in leisure-time physical activities, while other studies had information on specific types of physical activity (such as brisk walking, jogging, running, cycling, swimming, football). As our main aim was to evaluate the associations between physical inactivity and dementia, we constructed a measure of physical inactivity defined as no or very little moderate or vigorous physical activity or exercise based on the best available information in each study. Examples of definitions of physical inactivity are “less than 0.5 hour of each (brisk walking, jogging, or running) per week,” “no or very little exercise, only occasional walks,” and “sport activities a few times per year or less.” The definitions of physical inactivity in each of the participating studies are included in the appendix (pages 2-5). In addition, for five cohorts in the IPD-Work consortium, a harmonised three-level variable (low, moderate, and high physical activity) was also available.^15^

Age, sex, ethnicity (white *v* non-white), education/socioeconomic status (SES; harmonised into high, intermediate, and low), and prevalent dementia and cardiometabolic disease (coronary heart disease, stroke, and diabetes) were also assessed at baseline. Prevalent cases were excluded from the analyses of relevant endpoints. Other baseline characteristics, treated as covariates, included body mass index (weight (kg)÷(height (m)^2^)) treated as a continuous variable, cigarette smoking (current, former, or never smoker), and alcohol consumption (none, moderate, or heavy).^16^

### Follow-up for dementia, Alzheimer’s disease, and cardiometabolic disease

Data on dementia status at follow-up was extracted from national hospital admissions and death registries and reimbursements for medical treatment of dementia, with any mention of dementia in diagnostic codes as described previously.^16^ The definition varied slightly between studies (appendix, pages 2-5). Dementias were defined using the *International Classification of Diseases*, 10th revision, (ICD-10) codes F00, F01, F03, G30, and G31, with earlier ICD codes converted to ICD-10 codes.^17^ ^18^ Codes F00 and G30 were used to define Alzheimer’s disease.

We selected three cardiometabolic outcomes (type 2 diabetes, coronary heart disease, and stroke) known to be related to physical inactivity^19^ ^20^ ^21^ ^22^ ^23^ ^24^ ^25^ as positive controls to evaluate the validity of our approach and to examine the trajectory from physical activity to incident cardiometabolic disease and subsequent dementia. We ascertained these diseases from linked electronic health records from hospital admission, discharge, and mortality registers and via reported physician or health professional diagnosis as described previously (appendix, pages 2-5).^13^ ^26^ ^27^ Briefly, incident type 2 diabetes was identified with the ICD-10 diagnostic code E11.^26^ For incident coronary heart disease, we included all myocardial infarctions that were recorded as ICD-10 I21-I22 and coronary deaths recorded as ICD-10 I20-I25.^13^ We defined incident stroke using ICD-10 codes I60, I61, I63, I64 (for 13 US open-access studies, only a broader definition including codes I60-I69 was available).^27^

### Patient involvement

This is a secondary analysis of pre-existing datasets. No patients were involved in setting the present research question, the outcome measures, or in developing plans for recruitment, design, or implementation of the study. No patients were asked to advise on the interpretation or writing up of results. The dissemination plan targets a wide audience, including members of the public, patients, health professionals, and experts in the specialty through various channels: written communication, events and conferences, networks and social media.

### Statistical analysis

Syntax and detailed description of the statistical analyses are provided in the appendix (pages 5-11). Briefly, each participant was followed from the date of physical activity assessment to the first record of dementia (or cardiometabolic disease of interest), death, or the end of follow-up. In analyses of the associations of physical inactivity with all-cause dementia, Alzheimer’s disease, and each cardiometabolic disease, we used a two-step approach including study-specific analyses with Cox regression in the first step and pooling the study-specific estimates with random-effects meta-analysis in the second.

Study-specific hazard ratios and their 95% confidence intervals were combined using Knapp-Hartung estimators for between-study variance (these estimates are reported in the text).^28^ For comparison, the same meta-analyses were run using DerSimonian-Laird estimators for between-study variance (the default method in many software packages; these estimates are reported in the appendix, pages 13-20).^29^ Two estimators were used because evidence from empirical and simulation studies suggests that the commonly used DerSimonian-Laird variance estimator can produce biased estimates, particularly in meta-analyses based on small numbers of studies with moderate to substantial heterogeneity,^29^ and the Knapp-Hartung estimator can be less biased and more efficient.^28^ We calculated I^2^ and τ to estimate relative and absolute heterogeneity, respectively, among the study-specific estimates (in both indices, higher values denote greater heterogeneity).^30^

We adjusted the hazard ratios for the association between physical inactivity and dementia and Alzheimer’s disease for age, sex, ethnicity, and education/socioeconomic status (minimally-adjusted), and for body mass index, smoking, and alcohol intake (multivariable-adjusted).

We examined whether the hazard ratio for physical inactivity was non-proportional over the follow-up using pooled individual-participant data from all cohort studies. Two approaches were applied: Cox regression stratified by follow-up period (0 to <5 years, 5 to <10 years, 10 to <15 years, ≥15 years) and flexible parametric proportional-hazards for censored survival data on a log cumulative hazard scale (appendix, page 6).^31^ ^32^

To address reverse causation bias, the analysis was performed separately for incident dementia during the first 10 years of follow-up (when physical inactivity assessment is likely to fall in the preclinical or prodromal stage of dementia) and incident dementia from year 10 onwards in those without a dementia diagnosis at year 10. The underlying assumption in the second set of analyses (at least 10 years separating physical inactivity assessment and dementia diagnosis) is that the physical inactivity-dementia association is less likely to be biased by reverse causation. The 10 year threshold was chosen because studies with repeat measurements suggest physical activity in people with dementia begins to decline approximately a decade before diagnosis.^12^ For comparison, similar analyses were performed for each cardiometabolic disease.

To examine the robustness of the findings, we performed pre-selected subgroup analyses by sex, age (threshold 60 years), study-specific physical inactivity prevalence (threshold 40%), and method used for outcome ascertainment (electronic records from morbidity registers, mortality registers, or both). Due to smaller sample sizes in these subgroups, the analyses were based on pooled data across all cohorts rather than meta-analysis of study-specific estimates and were adjusted for study in addition to other covariates.

We also performed several other sensitivity analyses. We assumed that the long term level of physical activity has an impact on disease processes. As the value of a single measurement of physical activity reflects both the usual level and random fluctuations unrelated to disease processes, it will yield an underestimation of the true impact of physical inactivity on dementia. To address this potential source of bias, we corrected the hazard ratios using the Rosner method.^33^ To address potential survival bias, we conducted a Fine and Gray competing risk analysis with dementia and death as outcomes.^34^ To set the age of disease onset for cardiometabolic disease the same as that for dementia (≈80 years), we repeated the analysis of physical inactivity, incident diabetes, coronary heart disease, and stroke in a subgroup of participants who were alive and free of these diseases at age 65. To assess dose-response pattern, we used a three-level physical activity measure as the exposure.

Finally, to assess the association of physical inactivity with dementia in relation to cardiometabolic disease (that is, having one or more of diabetes, coronary heart disease, and stroke), we created two dementia endpoints for participants with no cardiometabolic disease at baseline and no dementia at year 10: (*a*) incident cardiometabolic disease followed by incident dementia and (*b*) incident dementia without preceding cardiometabolic disease. We tested whether physical inactivity was differently associated with these outcomes using the χ^2^ test (see appendix, pages 7-8).^35^ In these analyses, pooled data were used.

We used SAS (version 9.4) to analyse associations between physical inactivity and health outcomes separately in study-specific data. Stata (version 15) was used in flexible parametric proportional-hazards models and R (version 3.3.1) for meta-analyses combining study-specific estimates.

## Results

**Fig 1:**
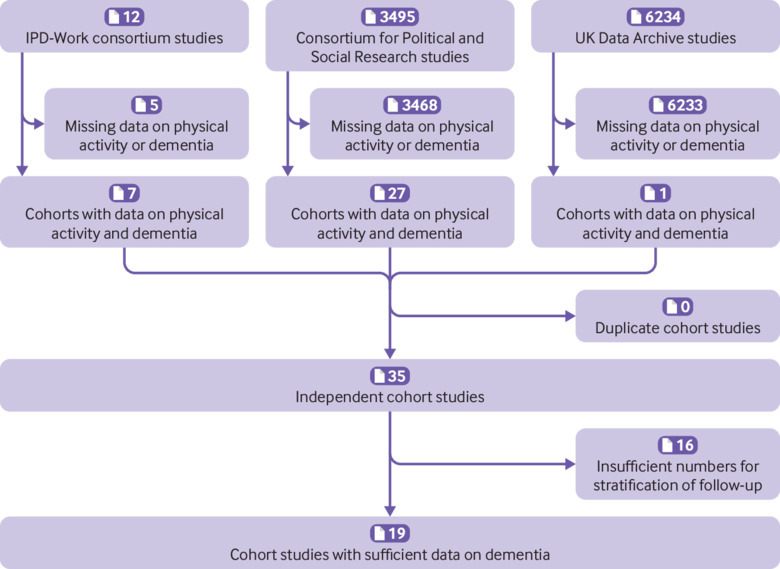
Selection of studies for individual-participant meta-analysis of the association between physical inactivity and dementia and Alzheimer’s disease

Of the 9741 studies identified in the three data sources, 35 had a measure of physical activity at baseline and follow-up for dementia (fig 1). In 19 of these studies, the length of follow-up and the number of incident dementia cases were sufficient for analysis of dementia risk within the first 10 years and from year 10 onwards. Fourteen studies used only death certificates to ascertain dementia, and five studies had dementia ascertainment based on electronic records from multiple registers including hospitalisations and medical prescriptions in addition to mortality records.

Of the 404 840 participants included, 171 336 were men and 233 504 women. Mean age at study entry was 45.5 years (range of mean ages across cohorts 36.7 to 54.3), and the prevalence of physical inactivity was 40.5% (appendix, page 12). Mean duration of follow-up for dementia was 14.9 years (range across studies 9.2 to 21.6 years). Over the 6 019 634 person-years at risk, 2044 incident dementia cases were recorded. For Alzheimer’s disease, 1604 incident cases were recorded during 5 210 933 person-years (total n=354 143). In a preliminary analysis ignoring potential non-proportionality, the age, sex, ethnicity, and socioeconomic status/education adjusted hazard ratio for the association between physical inactivity versus physical activity and dementia was 1.16 (95% confidence interval 1.03 to 1.31) (see appendix, pages 13).

### Analysis of non-proportionality

**Fig 2:**
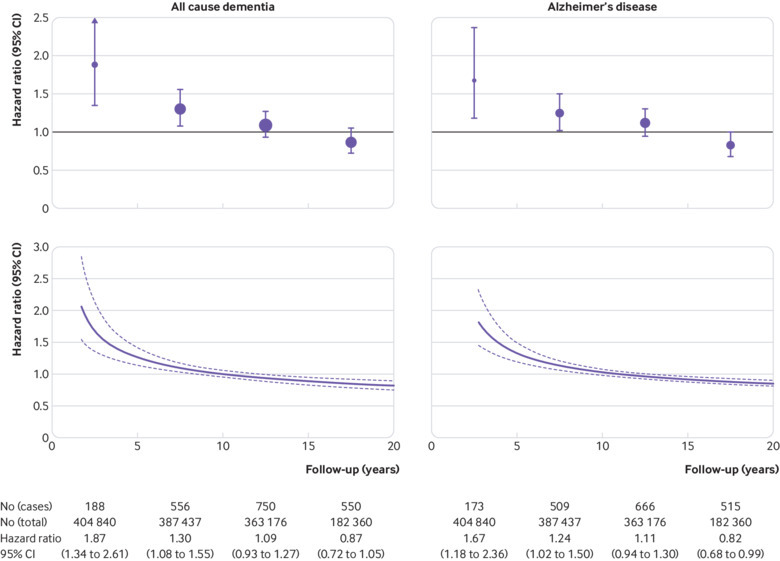
Change in hazard ratio for the association between physical inactivity at baseline (year 0) and risk of incident dementia and Alzheimer’s disease over the entire follow-up period (upper panel: follow-up-stratified analysis; lower panel: analysis of flexible parametric proportional-hazards)

As shown in figure 2, the associations of physical inactivity with dementia and Alzheimer’s disease varied over time, being strongest when the follow-up was short and attenuating to the null when follow-up was long. After adjustment for age, sex, ethnicity, and socioeconomic status/education, the hazard ratio for the association between physical inactivity and dementia was 1.87 (95% confidence interval 1.34 to 2.61) in years 0 to 4.9, 1.30 (1.08 to 1.55) in years 5-9.9, 1.09 (0.93 to 1.27) in years 10-14.9, and 0.87 (0.72 to 1.05) after year 15. The corresponding hazard ratios for Alzheimer’s disease were 1.67 (1.18 to 2.36), 1.24 (1.02 to 1.50), 1.11 (0.94 to 1.30), and 0.82 (0.68 to 0.99). This non-proportionality of hazards (departure from proportionality P<0.001 for dementia and Alzheimer’s disease) supported our decision to split follow-up period into two; the first 10 years of follow-up and from year 10 onwards.

### Association of physical inactivity with dementia in two follow-up periods

**Fig 3:**
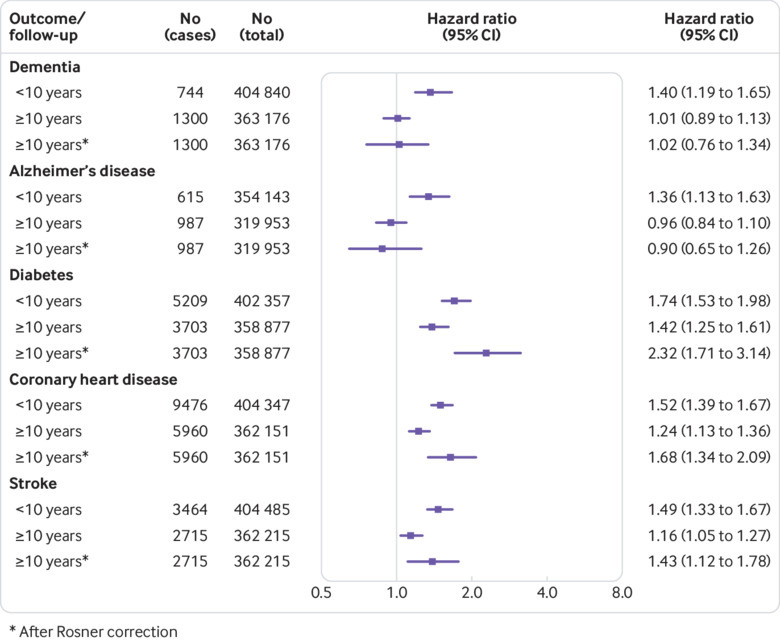
Summary hazard ratios from individual-level meta-analysis of 19 cohort studies for the association of physical inactivity with risk of dementia, Alzheimer’s disease, and cardiometabolic disease during the first 10 years of follow-up and from year 10 onwards in those without the disease at year 10

The age, sex, ethnicity, and socioeconomic status/education adjusted hazard ratio for the association between physical inactivity and dementia was 1.40 (95% confidence interval 1.24 to 1.59) for physical inactivity compared with physical activity when follow-up was <10 years and 1.01 (0.89 to 1.14) for dementia cases occurring after 10 years (an analysis addressing bias due to reverse causation) (fig 3, study-specific results in appendix, page 14). Further adjustment for smoking, alcohol consumption, and body mass index had little influence on the hazard ratios: 1.40 (1.21 to 1.62) and 1.02 (0.90 to 1.14) for the first and second parts of the follow-up. No heterogeneity in study-specific estimates was observed (I^2^=0%, τ=0, P=0.94 for the first follow-up period and I^2^=0%, τ=0, P=0.59 for the later period).

A similar pattern of results was seen for Alzheimer’s disease: the hazard ratio for the association between physical inactivity and incident Alzheimer’s disease was 1.36 (1.12 to 1.65) for a follow-up <10 years and 0.96 (0.85 to 1.08) for incident cases from year 10 onwards in those without a dementia diagnosis at year 10 (fig 3, I^2^=0%, τ=0, P=0.54 for the first follow-up period and I^2^=0%, τ=0, P=0.79 for the later period) (appendix, page 15).

**Fig 4:**
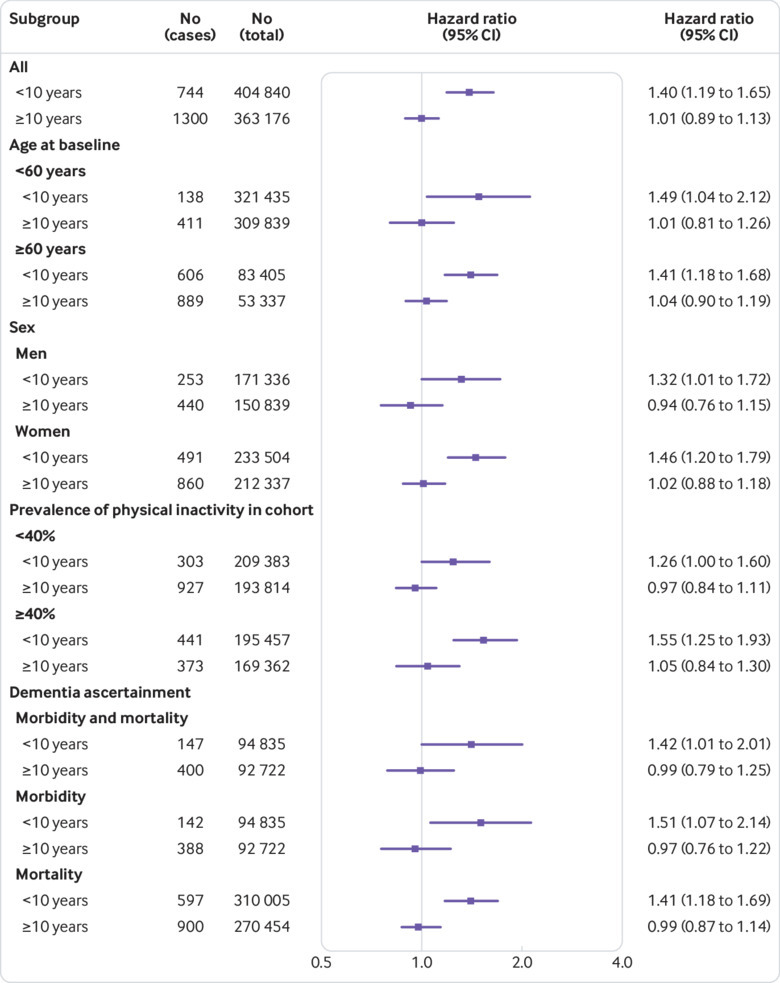
Hazard ratios for the association of physical inactivity with risk of dementia in subgroups from individual-level meta-analysis of 19 cohort studies

Figure 4 shows that these findings were robust, as the same difference in hazard ratios between the two follow-up periods was evident in men and women, and older and younger age groups, as well as in those studies in which the prevalence of physical inactivity was high or low. In participants above 60 years of age (mean age 70.8, standard deviation 7.6), for example, we recorded 606 dementia cases during the first 10 years of follow-up and 889 cases from year 10 onwards. The mean age at diagnosis was 84.8 years (standard deviation 6.7) during the first follow-up period and 86.7 years (SD 6.3) during the later period, and the hazard ratios for physical inactivity were 1.41 (1.18 to 1.68) and 1.04 (0.90 to 1.19), respectively.

The result was not affected by the method of dementia ascertainment (fig 4). With morbidity data (that is, ascertainment of dementia using linked records of hospitalisations and reimbursements for medical treatment of dementia), the hazard ratio for physical inactivity was 1.51 (1.07 to 2.14) in the first follow-up period and 0.97 (0.76 to 1.22) in the later period. The corresponding hazard ratios were 1.41 (1.18 to 1.69) and 0.99 (0.87 to 1.14) when only death records were available for ascertainment of dementia.

**Fig 5:**
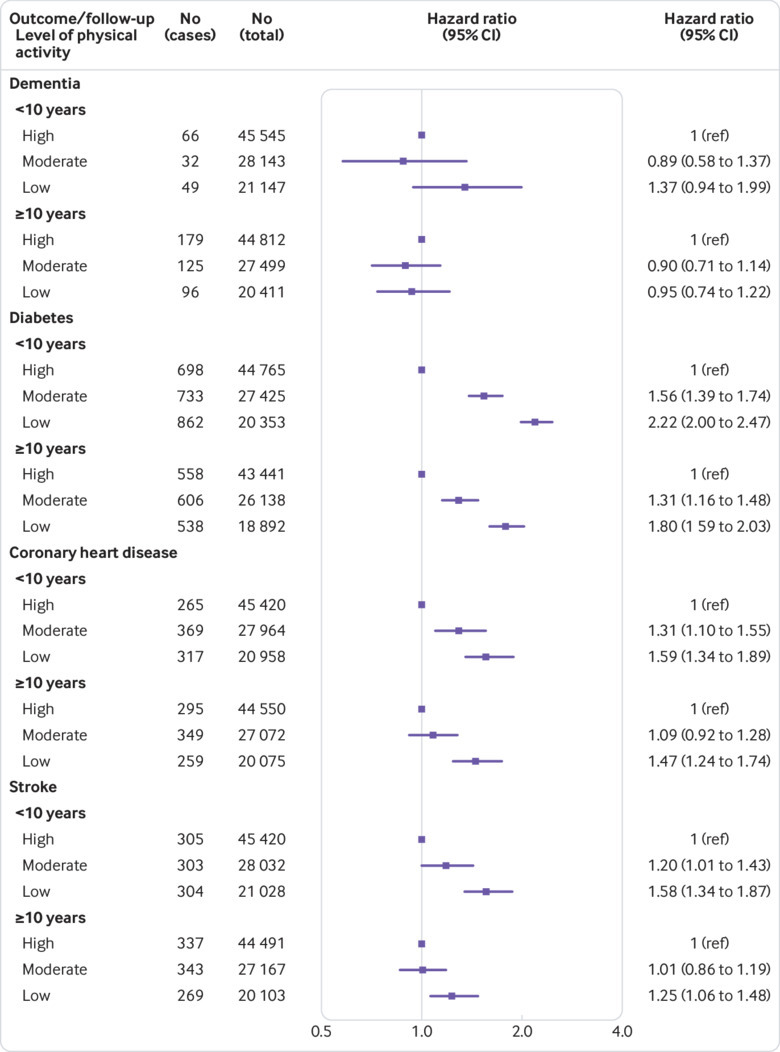
Hazard ratios from pooled analysis of 5 cohort studies for a 3-level physical activity in relation to dementia, diabetes, coronary heart disease, and stroke during the first 10 years of follow-up and from year 10 onwards in those without the disease at year 10

Sensitivity analyses addressing competing risk by mortality produced similar findings (appendix, page 19). Furthermore, the results did not change when the dichotomous physical activity variable was replaced with a more graded three-level variable in a subset of five cohorts (fig 5).

### Analysis of cardiometabolic disease as a positive control

Figure 3 shows that, in contrast to the dementia findings, physical inactivity was associated with an increased risk of incident diabetes, coronary heart disease, and stroke in both follow-up periods in models adjusted for age, sex, ethnicity, and socioeconomic status/education (appendix, pages 16-18). Correction for regression dilution in analyses with follow-up starting 10 years after the assessment of physical inactivity replicated this pattern of results. These findings suggest that the lack of association between physical inactivity and dementia in the later follow-up is not an artefact of the methodology.

In relation to all cardiometabolic diseases, there was evidence of a dose-response association in both parts of the follow-up period (fig 5). For example, the hazard ratios for intermediate and low physical activity compared with high physical activity were 1.56 (1.39 to 1.74) and 2.22 (2.00 to 2.47) in the first follow-up period and 1.31 (1.16 to 1.48) and 1.80 (1.59 to 2.03) in the later period. For coronary heart disease, the corresponding pairs of hazard ratios were 1.31 (1.10 to 1.55) and 1.59 (1.34 to 1.89), and 1.09 (0.92 to 1.28) and 1.47 (1.24 to 1.74). Other sensitivity analyses addressing the later onset of dementia relative to cardiometabolic disease also produced similar findings to the main analysis (appendix, page 11).

As shown in table 1, participants with prevalent diabetes, coronary heart disease, or stroke at baseline had an increased risk of developing dementia. The only exception was stroke, which, by causing immediate damage to the central nervous system, increased dementia risk soon after the event but not in those who were free from dementia at year 10. These expected findings support the validity of cardiometabolic disease and dementia ascertainment in this study.

**Table 1:** Associations of diabetes, coronary heart disease, and stroke at baseline with subsequent dementia (pooled analysis of five cohort studies)

Exposure at baseline, period of follow-up	No of dementia cases	Total No of participants	Hazard ratio (95% CI) for dementia
Diabetes (n=2196 exposed):			
All follow-up	546	94 739	1.57 (1.06 to 2.34)
Follow-up <10 years	147	94 739	1.61 (0.82 to 3.17)
Follow-up from year 10	399	92 638	1.55 (0.95 to 2.53)
Coronary heart disease (n=414 exposed):			
All follow-up	547	94 756	1.35 (0.60 to 3.04)
Follow-up <10 years	147	94 756	1.33 (0.33 to 5.42)
Follow-up from year 10:	400	92 646	1.34 (0.50 to 3.62)
Stroke (n of exposed=355)			
All follow-up	547	94 835	2.39 (1.07 to 5.34)
Follow-up <10 years	147	94 835	5.05 (1.86 to 13.7)
Follow-up from year 10	400	92 722	1.16 (0.29 to 4.64)
Any cardiometabolic disease* (n=2872 exposed):			
All follow-up	547	94 835	1.70 (1.21 to 2.37)
Follow-up <10 years	147	94 835	2.07 (1.20 to 3.55)
Follow-up from year 10	400	92 722	1.52 (0.99 to 2.32)

* Diabetes, coronary heart disease, or stroke

### Association of physical inactivity with dementia in relation to cardiometabolic disease

**Fig 6:**
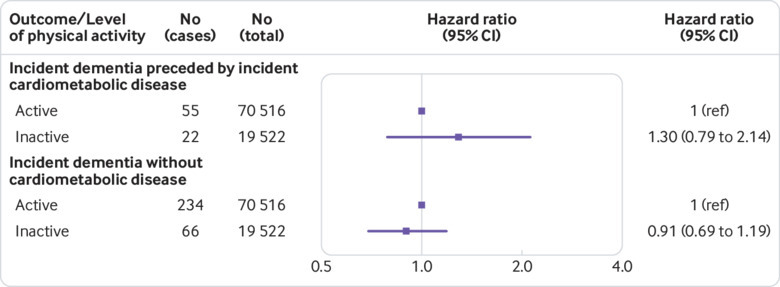
Hazard ratios from pooled analysis of 5 cohort studies for the association of physical inactivity with incident dementia with and without preceding incident cardiometabolic disease in participants with no cardiometabolic disease at baseline and no dementia at year 10

Morbidity and mortality data for disease trajectories were available from five studies (appendix, page 12) and a total of 94 835 participants, of whom 90 038 were free from cardiometabolic disease at baseline and had no history of dementia at year 10. Out of these 90 038 participants, 300 developed dementia without preceding incident cardiometabolic disease, and 77 first developed incident cardiometabolic disease (diabetes, coronary heart disease, or stroke) and then dementia. As shown in figure 6, there was an imprecisely estimated excess risk of dementia after cardiometabolic disease in physically inactive versus physically active individuals, the age, sex, ethnicity and socioeconomic status/education adjusted hazard ratio being 1.30 (0.79 to 2.14). No association was observed between physical inactivity and dementia with no preceding cardiometabolic disease (hazard ratio 0.91, 0.69 to 1.19). The difference between these two hazard ratios was not statistically significant at conventional levels (χ^2^(1)=1.56, P=0.21).

## Discussion

### Principal findings

In our multi-cohort analysis of individual-level data from 400 000 adults in Europe and the United States, there was no association between physical inactivity and dementia or Alzheimer’s disease when 10 years or more separated the assessment of physical inactivity and the dementia diagnosis (that is, when reverse causation bias is unlikely). In contrast, physical inactivity was associated with increased risk of incident diabetes, coronary heart disease, and stroke irrespective of the length of follow-up. There was some indication of a trajectory of risk from physical inactivity to cardiometabolic disease and subsequent dementia. However, this association was imprecisely estimated.

Prior longitudinal analyses with repeat data have shown that physical activity tends to decline in the preclinical or prodromal stage of dementia several years before clinical manifestation of symptoms; this is a major source of reverse causation bias in analyses based on short follow-ups.^12^ Accordingly, we found that low physical activity measured less than 10 years before dementia was linked to increased dementia incidence. In light of the previous findings of declining physical activity in the preclinical phase of dementia, this association may be biased.

### Comparison with other studies

The validity of our approach is supported by the fact that our observed associations between physical inactivity and dementia are similar to those from most recent literature-based meta-analyses. The summary effect estimate for physical inactivity was 1.4 (hazard ratio) in the present dataset for the first 10 years of follow-up and was 1.3 (odds ratio)^7^ and 1.4 (hazard ratio)^5^ in two previous meta-analyses of cohort studies in which most studies had a follow-up shorter than 10 years (13 of 16 and 19 of 21 studies, respectively). A slightly higher effect estimate, a hazard ratio of 1.5, was reported in a meta-analysis of people aged 70-80 years.^6^ This hazard ratio is similar to our findings for participants older than 60 in the <10 year follow-up. We believe these associations may be largely attributable to bias arising from changes in physical activity in the years preceding dementia and not from a true causal effect of physical activity on dementia risk.

The associations of diabetes, coronary heart disease, and stroke with incidence of dementia in our study are also close to those reported recently: the summary hazard ratio is 1.6 in our study versus 1.6 in previous meta-analyses for diabetes,^2^ 1.4 versus 1.3 for coronary heart disease,^3^ and 2.4 versus 1.7 for stroke.^4^ Furthermore, we found that physical inactivity was a risk factor for all three cardiometabolic diseases, in agreement with evidence-based clinical guidelines for prevention of type 2 diabetes, coronary heart disease, and stroke.^19^ ^20^ ^21^ ^22^ ^23^ ^24^ ^25^ The concordant effect estimates from our analysis and from previous investigations on physical inactivity and cardiometabolic diseases suggest that the characteristics of the cohorts or limitations in the assessment of physical inactivity are not an explanation for the lack of association between physical inactivity and dementia in our data.

In contrast to our analyses, some small-scale cohort studies reported long term associations between physical inactivity and risk of dementia, but these findings may be subject to type 1 error (false positive).^10^ ^36^ ^37^ ^38^ In a Finnish cohort study of 1250 adults, for example, physically active participants had a reduced risk of dementia (hazard ratio 0.47, 95% confidence interval 0.25 to 0.90), but, unexpectedly, the same participants were at increased risk of diabetes and there was no association of physical activity with blood pressure, total cholesterol, and body mass index.^10^ In a Japanese study of 803 community-dwelling elderly adults, physical inactivity was associated with increased risk of dementia (1.28, 0.99 to 1.67), but the association was stronger for Alzheimer’s disease than vascular dementia,^36^ although one would expect it to be the other way around. Because of their small size, adding the results from these two studies and from a further follow-up study reporting a non-significant positive association^37^ to our meta-analysis of the long term relation between physical inactivity and dementia (cases occurring after 10 years) does not alter our conclusion (summary hazard ratio 1.08, 0.95 to 1.23, for dementia and 1.12, 0.94 to 1.34 for Alzheimer’s disease; appendix, page 20).

### Strengths and limitations of study

Our study benefits from its large sample size, use of individual-level rather than study-level data in meta-analyses, and methodological triangulation in which multiple statistical approaches led to the same conclusion. Dementia ascertainment was based on electronic health records. This enabled all participants recruited to the study to be included in the analyses, rather than only those who continued to participate in follow-up examinations.^39^ ^40^

Some limitations to our study may have contributed to an underestimation of the effect of physical inactivity on dementia. We used a single, self reported measure of physical inactivity. This method is prone to reporting bias and does not capture cumulative effects of physical inactivity. We corrected for measurement error in physical inactivity using Rosner’s method, although this may not be sufficient to account for our crude assessment of the exposure. Ascertainment of dementia based on linkage to electronic health records is likely to miss milder cases of dementia.

However, several findings suggest that such crude exposure and outcome measures are capable of detecting associations when they exist.^21^ ^23^ ^41^ We observed the expected long term associations of physical inactivity as measured in this study with cardiometabolic disease. In addition, we demonstrated the expected associations of diabetes, coronary heart disease, and stroke with our outcome, incident dementia. For physical activity and dementia, no robust association was observed even when using an alternative, graded measure of physical inactivity, whereas long term dose-response associations were observed with all three cardiometabolic diseases.

The mean follow-up period for participating cohorts varied between nine and 21 years, with an overall mean of 15 years. This may not cover the entire preclinical phase for dementia as the first changes in dementia biomarkers are sometimes observed decades before clinical symptoms.^11^ However, previous studies with repeated physical activity measurements suggest that preclinical dementia only starts to affect levels of physical activity less than 10 years before the diagnosis of dementia.^12^

Residual confounding is possible. For example, data on high blood pressure, APOE e4 gene, depression, medication use, substance misuse, and pre-existing neurological disorders such as Parkinson’s disease and epilepsy were not available in all studies and could not be included as covariates in the analyses. However, unmeasured covariates, such as these, are an unlikely source of underestimation because, due to clustering of risk factors in the same individuals, they tend to inflate rather than mask associations.

Finally, with only 77 cases, our analysis of physical inactivity as a risk factor for a trajectory of incident cardiometabolic disease followed by incident dementia was underpowered. The imprecisely estimated 1.3-fold excess risk for this sequence of diseases among physically inactive participants is consistent with indirect evidence from our study and other investigations on physical inactivity as a risk factor for cardiometabolic disease^19^ ^20^ ^21^ ^22^ ^23^ ^24^ ^25^ and on cardiometabolic disease as a risk factor for dementia.^2^ ^3^ ^4^ If our findings and effect size were replicable, then 280 dementia cases with a history of cardiometabolic disease would be required in future studies to produce a statistically significant association between physical inactivity and incident cardiometabolic disease followed by dementia.

### Generalisability of the findings

We used cohort studies from different settings, but there was little heterogeneity in cohort-specific estimates for dementia, suggesting that our findings were generalisable to European and North American populations. Despite the relatively low mean age in the cohorts at study inclusion, our data are not limited to early, potentially more aggressive forms of the disease, as our main findings were replicated in a subgroup analysis of participants aged ≥60 years at baseline and with a mean age of 85 at dementia diagnosis.^42^

### Conclusion and policy implications

Physical activity is promoted as a simple, widely applicable, low cost strategy that could reduce the burden of diabetes, coronary heart disease and stroke.^19^ ^20^ ^21^ ^22^ ^23^ ^24^ ^25^ Our findings support this basic tenet of prevention in public health. However, there was little evidence that targeting physical inactivity alone would prevent dementia or Alzheimer’s disease. To confirm these findings, future large-scale studies should assess the cumulative amount of physical activity using repeated, ideally objective measures, such as wearable accelerometers, and extend dementia follow-up until old age or death for all participants.

**What is already known on this topic**

The status of physical inactivity as a risk factor for dementia is uncertain
Randomised controlled trials targeting physical inactivity show no evidence that it prevents or postpones dementia
Observational cohort studies may have overestimated dementia risk associated with physical inactivity as many studies are based on short follow-up times and thus subject to bias caused by decline in physical activity during the preclinical (prodromal) stage of dementia

**What this study adds**

In this meta-analysis of individual-level data from up to 400 000 adults, physical inactivity was associated with increased risk of incident diabetes, coronary heart disease, and stroke
When reverse causation bias was taken into account, physical inactivity was not associated with all-cause dementia or Alzheimer’s disease, although an indication of excess risk of dementia was observed in a subgroup of physically inactive individuals who developed cardiometabolic disease
These findings suggest that intervention strategies targeting physical inactivity alone will have limited effectiveness for dementia prevention

## Web Extra

Extra material supplied by the author

## Notes

Contributors: All authors participated in designing the study, generating hypotheses, interpreting the data, and critically reviewing the paper. MKi wrote the first draft of the report. Other members of the writing group were AS-M, SSa, JEF, GDB, and MJ. JP and JVL, with support from MJ and STN, analysed the data. MKi, JP, and MJ had full access to anonymised individual-participant data from all constituent studies. The corresponding author attests that all listed authors meet authorship criteria and that no others meeting the criteria have been omitted.

Ethical approval: Each cohort was approved by local ethics committees. Additional ethical approval for this meta-analysis is not required.

Data sharing: Syntax for data analysis is provided in the appendix. Our data protection agreements with the participating cohort studies do not allow us to share individual-level data from these studies to third parties.

Transparency: The lead author affirms that this manuscript is an honest, accurate, and transparent account of the study being reported; that no important aspects of the study have been omitted; and that any discrepancies from the study as planned (and, if relevant, registered) have been explained.

Dissemination plans. The dissemination plan targets a wide audience, including members of the public, patients, health professionals, and experts in the specialty through various channels: written communication, events and conferences, networks and social media.
