# Supplementary material for: Physical inactivity, cardiometabolic disease, and risk of dementia: an individual-participant meta-analysis
Source: BMJ. 2019 Apr 17;365:l1495. doi: 10.1136/bmj.l1495 (PMC6468884; doi:10.1136/bmj.l1495)
Supplement: Supplementary file 1 — Appendix [file kivm048363.w1.pdf]

APPENDIX

Contents

Expected association between physical inactivity and risk of dementia based on indirect evidence 2

Description of the cohort studies and definitions of physical inactivity and dementia 2

Statistical analysis 5

Statistical code 8

Supplementary results 11

eTable 1: Sample characteristics by cohort study 12

eFigure 1: Random-effects meta-analysis of the age, sex, ethnicity and socioeconomic status/education adjusted association between physical inactivity and all-cause dementia 13

eFigure 2: Random-effects meta-analysis of the age, sex, ethnicity and socioeconomic status/education adjusted association between physical inactivity and all-cause dementia with 2 follow-up periods 14

eFigure 3: Random-effects meta-analysis of the age, sex, ethnicity and socioeconomic status/education adjusted association between physical inactivity and Alzheimer's disease with 2 follow-up periods 15

eFigure 4: Random-effects meta-analysis of the age, sex, ethnicity and socioeconomic status/education adjusted association between physical inactivity and incident diabetes with 2 follow-up periods 16

eFigure 5: Random-effects meta-analysis of the age, sex, ethnicity and socioeconomic status/education adjusted association between physical inactivity and incident coronary heart disease with 2 follow-up periods 17

eFigure 6: Random-effects meta-analysis of the age, sex, ethnicity and socioeconomic status/education adjusted association between physical inactivity and incident stroke with 2 follow-up periods 18

eFigure 7: Summary estimates from age, sex, ethnicity and socioeconomic status/education associations of physical inactivity and dementia and Alzheimer's disease in a follow-up starting at year 10 before and after Fine & Gray correction for competing risk 19

eFigure 8: Random-effects meta-analysis of the long-term association between physical inactivity and all-cause dementia and Alzheimer's disease in the present data and those from previous studies 20

References 21

Expected association between physical inactivity and risk of dementia based on indirect evidence

Expected direct association between physical inactivity and risk of dementia based on previous studies on physical inactivity and risk of cardiometabolic disease and those on cardiometabolic disease and risk of dementia was calculated as follows:[1]

$$\text{Expected HR1} = e^{(\ln(HR2) * \ln(HR3))},$$

where HR1 is the expected hazard ratio for physical inactivity and dementia, HR2 is hazard ratio for physical inactivity and cardiometabolic disease, HR3 is hazard ratio for cardiometabolic disease and dementia, and ln is natural logarithm.

According to recent data syntheses and major studies, *HR2* for physical inactivity is approximately 1.2 in relation to diabetes,[2] 1.2 in relation to incident coronary heart disease,[3] and 1.2 in relation to stroke.[4] *HR3* for dementia as the outcome is 1.6 for diabetes,[5] 1.3 in relation to coronary heart disease,[6] and 2.2 in relation to stroke.[7]

Description of the cohort studies and definitions of physical inactivity and dementia

Finnish Public Sector study (FPS), Finland

The Finnish Public Sector study is a prospective cohort study comprising the entire public sector personnel of 10 towns (municipalities) and 21 hospitals in the same geographical areas. Participants, who were recruited from employers' records in 2000-2002, were individuals who had been employed in the study organisations for at least six months prior to data collection. 48 592 individuals (9 337 men and 39 255 women aged 17 to 65) responded to the questionnaire. Of these, 46,529 had data on physical activity and a follow-up of dementia and were eligible for our meta-analyses. Ethical approval was obtained from the ethics committee of the Finnish Institute of Occupational Health.

Physical inactivity was defined as less than 0.5 hour of each (brisk walking, jogging, or running) per week.

Participants were linked to drug reimbursement, hospitalisation and death registers. Dementia was defined using ICD-10, codes F00, F01, F02, F03, G30 and G31 (31.0, 31.1, 31.8)

Reference: Kivimäki M, Lawlor DA, Davey Smith G, et al. Socioeconomic position, co-occurrence of behavior-related risk factors, and coronary heart disease: the Finnish Public Sector study. *Am J Public Health* 2007; **97**: 874-9.

Gazel, France

Gazel is a prospective cohort study of 20 625 employees (15 011 men and 5 614 women) of France's national gas and electricity company, Electricité de France-Gaz de France (EDF-GDF). Since the study baseline in 1989, when the participants were aged 35–50 years, they have been posted an annual follow-up questionnaire to collect data on health, lifestyle, individual, familial, social, and occupational factors. Physical activity was measured in 1997 and 10,707 had data and were eligible for our meta-analysis. The GAZEL study received approval from the national commission overseeing ethical data collection in France (Commission Nationale Informatique et Liberté).

Physical inactivity was defined as “No sport activities“. For stroke ascertainment, only self-reports from annual follow-up surveys and mortality records were available. Dementia was defined using data from annual follow-up surveys requesting reported doctor-diagnosed demensis Alzheimer.

Reference: Goldberg M, Leclerc A, Bonenfant S, Chastang JF, Schmaus A, Kaniewski N, et al. Cohort profile: the GAZEL Cohort Study. *Int J Epidemiol* 2007; **36** :32-9.

Health and Lifestyle Survey (HALS)

UK HALS is a nationwide sample survey of community dwelling adults in England, Scotland, and Wales. In 1984/1985, a total of 12,254 addresses were randomly chosen from Electoral Registers and one adult aged 18 years or over was selected from each household. A total of 9003 adults participated in the baseline examination. Ethical approval for the main HALS surveys was received from the BMA Ethical Committee before the launch of survey. Physical activity was measured with a list of 17 different sports (e.g., cycling, swimming, football) and 4 open-ended sports the participant could select freely. Physical inactivity was defined as not participating in any sport activities.

Participants were linked to mortality registers and deaths from dementia were defined using ICD 9 209-294, 331.0, 331.1, 331.2, 331.8, and 331.9.

Reference: Cox BD, Blaxter M, Buckle ALJ, et al. The Health and Lifestyle Survey: A Preliminary Report. London: Health Promotion Trust; 1987.

Health and Social Support (HeSSup), Finland

The Health and Social Support (HeSSup) study is a prospective cohort study of a stratified random sample of the Finnish population in the following four age groups: 20–24, 30–34, 40–44, and 50–54. The participants were identified from the Finnish population register and posted an invitation to participate, along with a baseline questionnaire, in 1998. A total of 23,842 had data on physical activity and dementia and were thus eligible for our meta-analyses. The Turku University Central Hospital Ethics Committee approved the study.

Physical inactivity was defined as “less than 0.5 hour of each (brisk walking, jogging, or running) per week.” Participants were linked to drug reimbursement, hospitalisation and death registers. Dementia was defined using ICD-10, codes F00, F01, F02, F03, G30 and G31 (31.0, 31.1, 31.8).

Reference: Korkeila K, Suominen S, Ahvenainen J, Ojanlatva A, Rautava P, Helenius H, et al. Non-response and related factors in a nation-wide health survey. *Eur J Epidemiol* 2001; **17**: 991-9.

National Health and Nutrition Survey (NHANES) 1971, 1976, 1990, 1999, 2001, 2003, and 2005, USA

The National Health and Nutrition Examination Survey (NHANES) is a programme of studies designed to assess the health and nutritional status of adults in the United States. The original NHANES (original) sample included 20,729 persons 25 to 74 years of age. NHANES 1971 (original, n = 14 389), 1976 (n=9,235), 1990 (n=18,070), 1999 (n=5,432), 2001 (n=5,973), 2003 (n =5,605), and 2005 (n=5,556) are independent prospective cohort studies with data on physical activity and a follow-up for cause-specific deaths. In NHANES I, physical activity was measured with the question “Do you get much exercise in things you do for recreation?” with the response “little or no exercise” defined as physical inactivity (versus the responses “moderate exercise” and “much exercise”). In NHANES 2, the response options were the same but the question was “In things you do for recreation, for example, sports, hiking, dancing, and so forth, do you get much exercise, moderate exercise or little or no exercise?” with the response “little or no exercise” again defined as physical inactivity. In

NHANES III the participants were asked how often they participated in different sports included in a list of 9 physical activities (e.g., walking, jogging, swimming) and 1 option of other activity. Physical inactivity was defined as not participating in any of these activities. In the continuous NHANES cohorts from 1999 to 2005, the participants were asked two questions about their moderate and vigorous physical activities: (1) “Over the past 30 days, did you do any vigorous activities for at least 10 minutes that caused heavy sweating, or large increases in breathing or heart rate?” and (2) “Over the past 30 days, did you do moderate activities for at least 10 minutes that cause only light sweating or a slight to moderate increase in breathing or heart rate?” Physical inactivity was defined as answering no to both of these questions. Stroke was defined using a broader definition of ICD-10 codes I60-I69.

Available ICD codes for dementia as a cause of death varied between study baseline: For NHANES 1971, 1976 and 1988, dementia deaths were defined using ICD 9 codes: *209-294, 331.0, 331.1, 331.2, 331.8, 331.9.* For NHANES 1999 to 2005, dementia deaths were defined using ICD 10 G30 (Alzheimer’s disease).

References: Madans JH, Cox CS, Kleinman JC, et al. 10 years after NHANES I: mortality experience at initial follow up, 1982-84. *Public Health Rep* 1986; **101**: 474-81.

Flegal KM, Carroll MD, Kit BK, Ogden CL. Prevalence of obesity and trends in the distribution of body mass index among US adults, 1999-2010. *JAMA* 2012; **307**: 491-7.

Webpage: http://www.cdc.gov/nchs/nhanes/index.htm

National Health Interview Survey (NHIS) 1990-2009, USA

The National Health Interview Survey (NHIS) is a programme of studies monitoring the health of the U. S. residents since 1957. NHIS data on a broad range of health topics are collected through personal household interviews. The U.S. Census Bureau is the data collection agent for NHIS. For self-reported physical activity at baseline with a sufficient mortality follow-up for dementia deaths, data are publicly available from surveys in 1990 (n=39,434), 1991 (n=40,841), 1995 (n=16,234), 1997 (n=33,626), 1998 (n=29,815), 1999 (n=28,242), 2000 (n=29,501), 2001 (n=30,241), 2002 (n=27,968), 2003 (n= 27,338), 2004 (n=28,318), 2005 (n=28,167), 2006 (n=22,543), 2007 (n=21,892), 2008 (n=20,665), and 2009 (n=26,610). The participants were asked how often they performed moderate and vigorous physical activities. Physical inactivity was defined as participating in no leisure-time aerobic activity that lasted at least 10 minutes. Stroke was defined using a broader definition of ICD-10 codes I60-I69. In NHIS 1990 and 1995, data on alcohol consumption were not available. NHIS 1995 also lacked information about smoking habits.

Dementia was defined using death records, ICD-10 code G30 (Alzheimer’s disease).

Reference: Dawson DA. Ethnic-differences in female overweight - data from the 1985 National-Health Interview Survey. *Am J Public Health* 1988; **78**: 1326-29.

Webpage: http://www.cdc.gov/nchs/nhis/

Still Working

Still Working is an ongoing prospective cohort study. In 1986, the employees (n = 12,173) at all Finnish centres of operation of Enso Gutzeit (a forestry products manufacturer) were invited to participate in a questionnaire survey on demographic, psychosocial and health-related factors. Physical activity was measured at study baseline in 1986 and 9058 provided data and were followed

up for dementia. The study was approved by the ethics committee of the Finnish Institute of Occupational Health.

Physical inactivity was defined as ”Sport activities less than a couple of times per month.” Data on height and weight (BMI) were not available.

Participants were linked to drug reimbursement, hospitalisation and death registers. Dementia was defined using ICD-10, codes F00, F01, F02, F03, G30 and G31 (31.0, 31.1, 31.8)

Reference: Kalimo R, Toppinen S. Organizational well-being: ten years of research and development: in a forest industry corporation. In: Kompier M, Cooper C, editors. Preventing Stress, Improving Productivity: European Case Studies in the Workplace. London: Routledge; 1999. p. 52-85.

WOLF (Work, Lipids, and Fibrinogen) Norrland, Sweden

WOLF Norrland is a prospective cohort of 4,699 participants aged 19-65 working in companies in Jämtland and Västernorrland counties and with data on physical activity and dementia. At study baseline the participants underwent a clinical examination and completed a set of health questionnaires. The baseline assessment was undertaken at 13 occupational health service units in 1996-98. The Regional Research Ethics Board in Stockholm, and the ethics committee at Karolinska Institutet, Stockholm, Sweden approved the study.

Physical inactivity was defined as “No or very little exercise, only occasional walks.” Dementia was defined using ICD-10, codes F00, F01, F02, F03, G30 and G31 (31.0, 31.1, 31.8).

References: Peter R, Alfredsson L, Hammar N, Siegrist J, Theorell T, P. W. High effort, low reward, and cardiovascular risk factors in employed Swedish men and women: baseline results from the WOLF Study. *J Epidemiol Community Health* 1998; **52** :540-7

Alfredsson L, Hammar N, Fransson E, de Faire U, Hallqvist J, Knutsson A, et al. Job strain and major risk factors for coronary heart disease among employed males and females in a Swedish study on work, lipids and fibrinogen. *Scand J Work Environ Health* 2002; **28**: 238-48.

Statistical analysis

In the main analysis of all-cause dementia, Alzheimer’s disease and each cardiometabolic disease, we used a 2-step individual-participant-data meta-analysis including study-specific analyses in the first step and pooling the study-specific estimates in the second. In each study, we performed Cox regression to generate hazard ratios and accompanying 95% confidence intervals (CI) for the association between physical inactivity (yes vs no) and the outcomes. Each participant was followed up from the date of physical inactivity assessment to the first record of dementia (or cardiometabolic disease of interest), death, or the end of follow-up.

To take into account that the associations are not necessarily similar across different settings, we present the summary hazard ratios from the random-effects models. Study-specific hazard ratios and their 95% confidence intervals (CIs) were combined using Knapp-Hartung estimator for between-study variance (these estimates are reported in text).[8] For comparison, the same meta-analyses were run using DerSimonian-Laird estimator for between-study variance (the default method in many software packages; these estimates are reported in appendix).[9] Two estimators were used because evidence from empirical and simulation studies suggests that the

commonly used DerSimonian-Laird variance estimator can produce biased estimates particularly in meta-analyses based on small numbers of studies with moderate to substantial heterogeneity,[9] and Knapp-Hartung estimator can be less biased and more efficient.[8] We calculated I^2 and τ to estimate relative and absolute heterogeneity, respectively, among the study-specific estimates (in both indices, higher values denote greater heterogeneity).[10]

We adjusted the hazard ratios for physical inactivity for age, sex, ethnicity, education/socioeconomic status (minimally-adjusted), and additionally BMI, smoking, and alcohol intake (multivariable-adjusted). We included in the analysis participants without missing data on the exposure, covariates in the minimally-adjusted model and outcome, but imputed missing covariates for BMI, smoking and alcohol intake, if the missingness was >10% (multivariate stochastic imputation with chained equations). In a preliminary analysis, we examined the association between physical inactivity and dementia ignoring potential non-proportionality; this approach corresponds to meta-analyses that are possible to conduct using only summary data from published studies.

We then examined whether the hazard ratio for physical inactivity was non-proportional over the follow-up using pooled individual-participant data from all cohort studies (these analyses were additionally adjusted for cohort). Two approaches were applied: Cox regression stratified by follow-up period (0 to <5 years, 5 to <10 years, 10 to <15 years, ≥15 years) and flexible parametric proportional-hazards for censored survival data on a log cumulative hazard scale.[11] In the latter analysis, we used the Akaike information criterion[12] to assist selection of the parametric model (the final model had two degrees of freedom for the restricted cubic spline function used for the baseline hazard rate and 1 degree of freedom for time-dependent effect of physical activity).

The analysis was then performed separately for incident dementia during the first 10 years of follow-up (when most dementia cases were expected to be at the preclinical or prodromal stage of dementia at the time of baseline physical inactivity measurement) and incident dementia from year 10 onwards for those who did not have dementia at year 10. As in the latter analysis the physical inactivity assessment was 10 years or more before recorded dementia, we assumed it was less likely affected by preclinical/prodromal stage of dementia. Similar analyses were performed for each cardiometabolic disease. The estimates for physical inactivity were adjusted for age (continuous variable), sex, ethnicity (white vs other) and education/socioeconomic status (high, intermediate, low). Multivariable-adjusted effect estimates were additionally adjusted for BMI (continuous variable), smoking status (current, ex-, never smoker), and alcohol intake (none, moderate, high).

Our analysis with follow-up starting from year 10 onwards is subject to regression dilution bias as people may change their physical activity during the first 10 years of follow-up. We assumed that the long-term level of physical activity has an impact on disease process. As the value of a single measurement of physical activity reflects both the usual level and random fluctuations unrelated to disease process, it will yield an underestimation of the true impact of physical inactivity on dementia. To address this potential source of bias, we corrected hazard ratios for regression dilution using Rosner method[13] and information from the stability of physical activity. The latter was estimated from repeated measurements of physical activity in two cohort studies with repeat physical inacitivty assessment (the Finnish Public Sector study and the Health and Social Support Study) and the pooled correlation coefficient was r=0.416 for 10-year stability.

For comparison to our analyses of physical inactivity and dementia, we examined the associations of physical inactivity with incident diabetes mellitus, coronary heart disease and stroke and treated them as positive controls. We assumed that if the positive control does not produce the

expected result (i.e. an association of physical inactivity with incident type 2 diabetes, coronary heart disease and stroke is observed as these associations have previously been confirmed in meta-analyses of cohort studies and using randomized controlled trials on these or surrogate outcomes),[5-7] our measurement of physical inactivity or analytic procedure might not be correct and thus also findings of dementia might not be valid. Expected associations between physical inactivity and these cardiometabolic diseases, in turn, support the validity of our analytic approach.

To assess dose-response pattern, we repeated the main analyses using a 3-level physical activity measure as the exposure. This measure was available from the Finnish Public Sector study (FPS), the Health and Social Support study (HeSSup), WOLF, GAZEL, and the Still Working study.

To examine robustness of our findings, we performed pre-selected subgroup analyses by sex, age (threshold 60 years), study-specific physical inactivity prevalence (threshold 40%) and outcome ascertainment method (electronic records from morbidity registers, mortality registers, and both). Due to smaller sample sizes in these subgroups, the analyses were based on pooled data across all cohorts rather than meta-analysis of study-specific estimates and were adjusted for study in addition to other covariates.

To address potential survival bias we conducted a Fine and Gray competing risk analysis with dementia and death as outcomes.[14] This analysis was confined to the 5 studies with dementia identified using morbidity and mortality data. The late onset of disease may introduce survival bias. As the onset of dementia is at an older age than those of diabetes and coronary heart disease, we repeated the analysis of physical inactivity, diabetes, coronary heart disease and stroke in participants who were alive at age 65 and free of the health outcome of interest at that age. In this group, mean age at recorded incident diabetes, coronary heart disease or stroke was comparable to the mean age of recorded dementia in the entire cohort, thus reducing any differences in survival bias between analyses of dementia and the other health outcomes. The purpose of this sensitivity analysis was to compare the association between physical inactivity and cardiometabolic diseases to that between physical inactivity and dementia when the age of disease onset is the same for cardiometabolic diseases and dementia.

To assess the association of physical inactivity with dementia in relation to cardiometabolic disease (i.e. having one or more of diabetes, coronary heart disease and stroke), we formed two dementia endpoints for participants with no cardiometabolic disease at baseline and no dementia at year 10:

(1) incident cardiometabolic disease followed by incident dementia (defined as cases, all others as non-cases) and

(2) incident dementia without preceding cardiometabolic disease (defined as cases, all others as non-cases).

Censoring was at the date of dementia, death or end of follow-up. We tested whether physical inactivity was differently associated with these outcomes using the following formula:

χ^2(1 degree of freedom) = $(b_1-b_2)^2/(SE_1^2+SE_2^2)$,
where b_i is parameter estimate for event endpoint i
SE_i is standard error for event endpoint i.[15]

In these analyses, pooled data were used. Morbidity and mortality data for these disease trajectories were available from 5 studies: the Finnish Public Sector study (FPS), the Health and Social Support study (HeSSup), WOLF, GAZEL, and the Still Working study.

We used SAS (version 9.4) to analyse physical inactivity-health outcome associations separately in study-specific data. R (version 3.3.1) was used in meta-analyses to combine study-specific estimates.

Statistical code

***Imputation** (stata code)*

```
cap mi unset
mi set flong
mi register imputed SES bmi alcocl smokerex edu1
mi register regular sex age1 inactive status_mort
mi impute chained (reg) bmi (pmm, knn(5)) alcocl (ologit) SES (mlogit)
smokerex  = sex age1 inactive status_mort ethnicity, add(1) rseed(984571)
noisily
```

Figure 2:

Stata code (for both all-cause dementia and Alzheimer's disease as outcome):

```
stpm2 age sex i.ses inactive, df(2) scale(hazard) tvc(inactive) dftvc(1)
eform
est store model1
predict hr, hrnum(inactive 1) hrdenom(inactive 0) timevar(_t) ci
estimates stats model1
```

SAS code:

```
proc phreg data=yht2;
class inactive(ref='0') SES ethnicity study;
* <5 yrs     *;
model futimeA1*statusA1(0)= sex age SES ethnicity study inactive / rl;
* 5- <10 yrs *;
*model futimeA2*statusA2(0)= sex age SES ethnicity study inactive / rl;
* 10- <15 yrs*;
*model futimeB1*statusB1(0)= sex age SES ethnicity study inactive / rl;
* 15- yrs    *;
*model futimeB2*statusB2(0)= sex age SES ethnicity study inactive / rl;
run;
```

Figure 3 & Appendix Figures 1-6,8:

SAS code:

```
proc phreg data=yht2;
class inactive(ref='0') SES ethnicity;
*class inactive(ref='0') SES ethnicity alcocl smokerex;
model futime*status(0)= sex age SES ethnicity inactive / rl; * all *;
*model futimeA*statusA(0)= sex age SES ethnicity inactive / rl; * <10yrs*;
*model futimeB*statusB(0)= sex age SES ethnicity inactive / rl; * 10+yrs*;
*model futime*status(0)= sex age SES ethnicity bmi alcocl smokerex inactive
/ rl; * all, multivariable adjusted *;
by study;
ods output ParameterEstimates=pe CensoredSummary=cs;
data pe; set pe; if parameter='inactive';
data res; merge pe cs; by study;
```

```
keep study Estimate StdErr ProbChiSq HazardRatio HRLowerCL HRUpperCL Total
Event;
proc print data=res;
run;
```

R code:

Knapp-Hartung estimator

```
library(metafor)

labels<-c("FPS","Gaz","HALS","HEA","STW","WON","nhanes1971","nhanes1999",
"nhanes2","nhanes2001","nhanes3","nhis1990","nhis1991","nhis1995","nhis1997
","nhis1998","nhis1999","nhis2000","nhis2001")

est1<-c (0.08479,-0.01386,0.36011,0.18616,0.06001,0.49915,0.03004,0.43480,-
0.85263, 0.29342,0.38270,0.07668,-0.19465,0.14180,-
0.02180,0.30499,0.30070,0.59093,0.73747)

var1<-c
(0.12872^2,0.57401^2,0.35944^2,0.25062^2,0.20772^2,0.71187^2,0.20324^2,0.46
894^2,
0.58231^2,0.35423^2,0.23621^2,0.13050^2,0.12676^2,0.22601^2,0.17415^2,0.204
34^2,0.21882^2,0.24537^2,0.26525^2)

kh<-rma.uni(est1,var1,measure="RR",method="DL",knha=TRUE)
summary(kh)
forest(kh,atransf=exp)
```

Appendix Figure 7:

SAS code:

```
* morbidity, competing risk, 10+ yrs *;
proc phreg data=yht2; where IPD=1;
class inactive(ref='0') SES ethnicity study;
model futime_dem2B*status_dem3B(0)= sex age SES ethnicity study inactive /
eventcode=1 rl;
run;
```

Figure 4:

SAS code:

```
proc phreg data=yht2;
where age2=1;
*where age2=2;
*where sex=1;
*where sex=2;
*where palevel=1;
*where palevel=2;
class inactive(ref='0') study SES ethnicity;
model futimeA*statusA(0)= sex age SES ethnicity study inactive / rl; * <10
yrs*;
*model futimeB*statusB(0)= sex age SES ethnicity study inactive / rl; * 10+
yrs*;
run;
proc phreg data=yht2; where IPD=1;
class inactive(ref='0') study SES ethnicity;
```

```
model futimeA*statusA(0)= sex age SES ethnicity study inactive / rl; *
<10*;
*model futimeB*statusB(0)= sex age SES ethnicity study inactive / rl; *
10+*;
run;
proc phreg data=yht2; where IPD=1;
class inactive(ref='0') SES study;
model futime_dem2A*status_dem2A(0)= sex age SES study inactive / rl; *
morbidity, <10*;
*model futime_dem2B*status_dem2B(0)= sex age SES stud2 inactive / rl; *
morbidity, 10+*;
run;
proc phreg data=yht2; where IPD=0;
class inactive(ref='0') study SES ethnicity;
model futimeA*statusA(0)= sex age SES ethnicity study inactive / rl; *
mortality, <10*;
*model futimeB*statusB(0)= sex age SES ethnicity study inactive / rl; *
mortality, 10+*;
run;
```

Figure 5:

SAS code:

```
* physical activity (0-2) *;
proc phreg data=yht2;
class study SES ethnicity h_physact(ref='2');
model futimeA*statusA(0)= sex age SES ethnicity study h_physact / rl;
*model futimeB*statusB(0)= sex age SES ethnicity study h_physact / rl;
run;
```

Figure 6:

SAS code:

```
data yht3;
set yht2;
IF IPD=1 and exdisease=0;
status_dis=max(status_db,status_chd,status_stroke);
futime_dis=min(futime_db,futime_chd,futime_stroke);
if status_dis=1 and statusB=1 and futime_dis>futimeB then status_dis=0;
statusB_dis=statusB; if statusB=1 and status_dis=0 then statusB_dis=0;
statusB_nodis=statusB; if statusB=1 and status_dis=1 then statusB_nodis=0;
run;
proc phreg data=yht3;
class inactive(ref='0') study SES ethnicity;
* 10+ yrs *;
model futimeB*statusB_dis(0)= sex age SES ethnicity study inactive / rl;
*model futimeB*statusB_nodis(0)= sex age SES ethnicity study inactive / rl;
run;
```

Table 1:

SAS code:

```
proc phreg data=yht2; where IPD=1; ** exposures:
exdb,exchd,exstroke,exdisease **;
class exdb(ref='0') study SES ethnicity;
model futime*status(0)= sex age SES ethnicity study exdb / rl; * all *;
*model futimeA*statusA(0)= sex age SES ethnicity study exdb / rl; * <10 *;
```

```
*model futimeB*statusB(0)= sex age SES ethnicity study exdb / rl; * 10+ *;
run;
```

Supplementary results

eTable 1 reports descriptive data for the included cohort studies. **eFigure 1** shows results from the random-effect meta-analysis on the association between physical inactivity and dementia in the entire follow-up. Results from meta-analyses of the associations between physical inactivity and disease insidence in two different follow-ups are shown in **eFigures 2-6** for incident dementia, Alzheimer's disease, diabetes, coronary heart disease and stroke.

In relation to the follow-up from year 10 onwards for incident dementia, hazard ratios favoured risk factor status for physical inactivity in 12 studies and a protective effect in 7 studies. In this latter follow-up for Alzheimer's disease, 6 hazard ratios favoured risk status and 7 a protective effect. In contrast, despite heterogeneity in study-specific estimates, the hazard ratios for physical inactivity favoured risk factor status in 16-17 of the 18 studies on diabetes in both follow-ups. This was the case for 18-19 of the 19 studies on incident coronary heart disease and 15-18 of the 19 studies on incident stroke.

The mean age at ascertainment of dementia was high (80.6 years), compared to the mean age for diabetes, coronary heart disease and stroke (66.8, 75.1 and 73.4 years), raising the possibility of more severe survival bias as an explanation for weaker associations of physical inactivity in relation to dementia compared to cardiometabolic disease. This possibility was not supported by Fine and Gray competing risk analyses for dementia (**eFigure 7**), or a sensitivity analysis of incident cardiometabolic disease in a group of participants without cardiometabolic disease at age 65. In the latter analysis, the excess risk of diabetes, coronary heart disease and stroke associated with physical inactivity was evident (hazard ratios 1.27, 95% CI 1.16-1.39; 1.14, 95% CI 1.07-1.21; and 1.07 95% CI 0.97-1.17, respectively) in spite of the mean age at ascertainment of the health outcome being comparable to that for dementia (77.9, 81.9 and 80.5, respectively).

eFigure 8 shows that adding previous long-term follow-up studies[16-18] to our meta-analysis of the association between physical inactivity and dementia when follow-up for dementia in our studies was started at year 10 did not change conclusions from our main analysis.

eTable 1. Cohort characteristics

Study	Baseline	N(Total)	Mean age at baseline	Proportion of women	Proportion of inactive	Mean follow-up, years	N(dementia)	N(Alzheimer's disease)	Method of dementia ascertainment	N(diabetes)	N(coronary heart disease)	N(stroke)
Nhanes1971	1971	14,389	48.9	59.7	44.3	18.1	102		Deaths	97	1297	405
Nhanes2	1976	9,235	54.3	53.0	41.4	13.3	16		Deaths	36	565	134
HALS	1984	9,003	45.9	56.6	58.4	20.8	57		Deaths	42	711	343
Still Working	1986	9,058	40.9	22.3	19.5	21.6	138	103	Hospital, prescriptions, deaths	713	712	466
Nhanes3	1990	18,070	46.9	53.3	26.9	13.2	86		Deaths	148	435	347
Nhis1990	1990	39,403	44.8	58.1	34.1	19.2	247	247	Deaths	918	2406	719
Nhis1991	1991	40,841	44.8	57.8	37.1	18.6	270	270	Deaths	912	2231	659
Nhis1995	1995	16,234	45.3	56.6	36.0	15.4	80	80	Deaths	279	686	192
WOLF N	1996	4,699	44.1	16.7	26.8	12.6	9	2	Hospital, deaths	47	133	95
Gazel	1997	10,707	50.3	27.4	37.7	13.7	13	13	Self-reports, deaths	673	258	299
Nhis1997	1997	33,626	46.1	57.3	54.9	13.9	154	154	Deaths	631	1302	388
HeSSup	1998	23,842	36.7	59.1	20.5	14.8	81	45	Hospital, prescriptions, deaths	888	238	279
Nhis1998	1998	29,815	46.5	56.3	55.7	13.0	128	128	Deaths	510	1099	307
Nhanes1999	1999	5,432	47.1	53.3	51.0	10.8	25	25	Deaths	49	213	60
Nhis1999	1999	28,242	46.7	57.3	55.3	12.3	109	109	Deaths	459	899	255
FPS	2000	46,529	44.6	80.7	19.7	15.5	306	203	Hospital, prescriptions, deaths	1674	513	722
Nhis2000	2000	29,501	46.4	57.0	54.0	11.4	95	95	Deaths	409	866	249
Nhanes2001	2001	5,973	46.9	52.6	43.8	9.2	38	38	Deaths	27	161	72
Nhis2001	2001	30,241	46.3	56.6	53.3	10.6	90	90	Deaths	400	711	188
Total		404,840	45.5	57.7 (233 504)	40.5	14.9	2,044	1,602		8,912	15,436	6,179

All-cause dementia

Study	Minimally adjusted N(cases)	N(total)	HR (95% CI)	Multivariable adjusted* N(cases)	N(total)	HR (95% CI)
WOLF N	9	4699	1.65 (0.41 to 6.65)	9	4453	1.88 (0.46 to 7.69)
Nhanes2*	16	9235	0.43 (0.14 to 1.34)	16	9235	0.47 (0.15 to 1.49)
Gazel	13	10707	0.99 (0.32 to 3.04)	13	10330	0.95 (0.30 to 2.97)
Nhanes1999*	25	5432	1.55 (0.62 to 3.87)	21	4897	1.64 (0.60 to 4.45)
HALS*	57	9003	1.43 (0.71 to 2.90)	56	8888	1.29 (0.64 to 2.62)
Nhanes2001*	38	5973	1.34 (0.67 to 2.69)	38	5386	1.22 (0.60 to 2.47)
Nhis2001*	90	30241	2.09 (1.24 to 3.52)	88	29222	2.14 (1.26 to 3.64)
HeSSup	81	23842	1.21 (0.74 to 1.97)	75	21779	1.14 (0.67 to 1.93)
Nhis2000*	95	29501	1.81 (1.12 to 2.92)	93	28535	1.79 (1.10 to 2.91)
Nhanes3	86	18070	1.47 (0.92 to 2.33)	72	16794	1.16 (0.68 to 1.97)
Nhis1995	80	16234	1.15 (0.74 to 1.80)	79	15930	1.17 (0.75 to 1.82)
Nhis1999*	109	28242	1.35 (0.88 to 2.07)	104	27417	1.28 (0.83 to 1.98)
Still Working	138	9058	1.06 (0.71 to 1.60)	138	8957	1.02 (0.68 to 1.55)
Nhis1998*	128	29815	1.36 (0.91 to 2.03)	124	29058	1.33 (0.88 to 1.99)
Nhanes1971*	102	14389	1.03 (0.69 to 1.54)	102	14382	0.99 (0.67 to 1.48)
Nhis1997*	154	33626	0.98 (0.70 to 1.38)	151	32831	1.09 (0.76 to 1.55)
Nhis1990	247	39403	1.08 (0.84 to 1.39)	243	38855	1.13 (0.87 to 1.46)
FPS	306	46529	1.09 (0.85 to 1.40)	285	44382	1.09 (0.83 to 1.42)
Nhis1991	270	40841	0.82 (0.64 to 1.06)	260	39589	0.88 (0.68 to 1.13)
**Knapp-Hartung estimate**			1.16 (1.03 to 1.31)			1.13 (1.02 to 1.26)
**DerSimonian-Laird estimate**			1.16 (1.03 to 1.30)			1.13 (1.03 to 1.25)

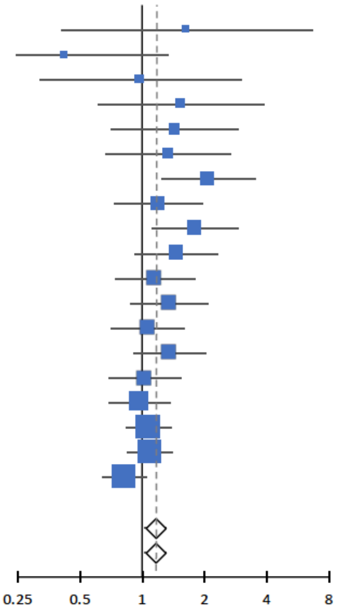

Random effects model
Heterogeneity: I²=23%, τ=0.12, p=0.1731

Minimally adjusted hazard ratios are adjusted for age, sex, ethnicity and SES/education
Multivariable adjusted hazard ratios are adjusted for age, sex, ethnicity, SES/education, BMI, alcohol consumption and smoking
*Missing data on BMI, alcohol consumption and smoking imputed (multivariate stochastic imputation with chained equations)

eFigure 1. Hazard ratio for the association of physical inactivity with risk of all-cause dementia after minimal adjustment (age, sex, ethnicity and SES/education as covariates) and multivariable adjustment (smoking, BMI, physical activity, and alcohol consumption as additional covariates).

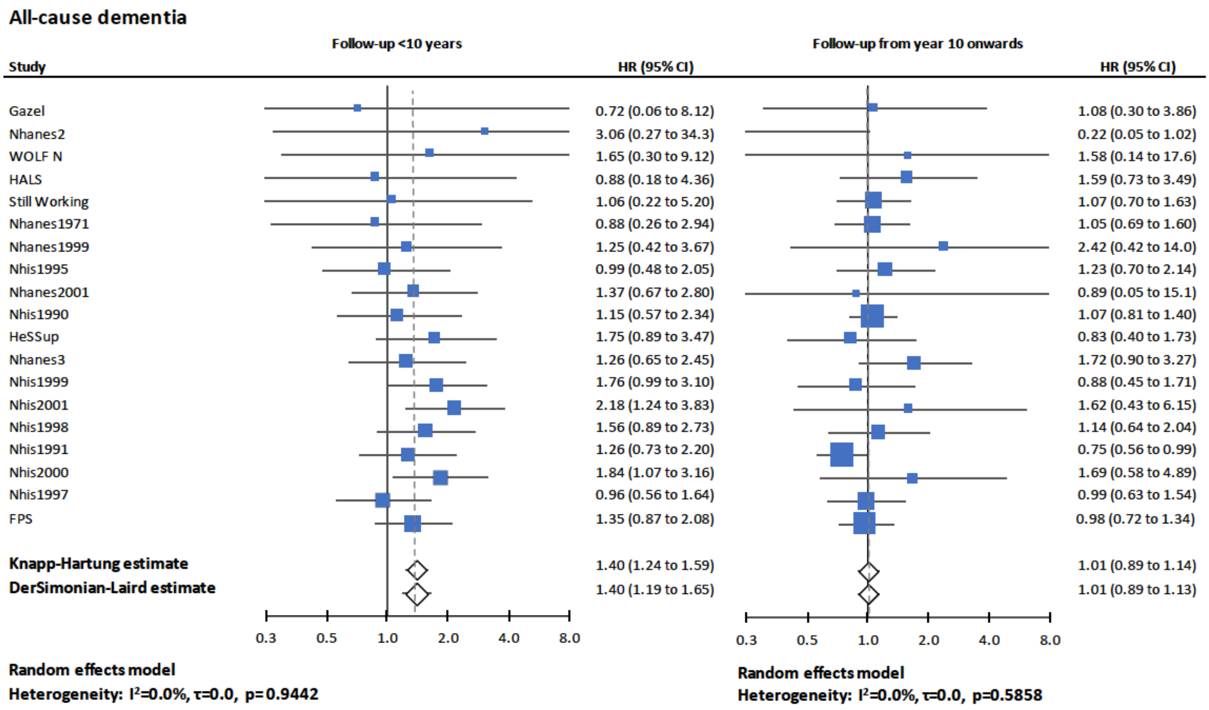

eFigure 2. Hazard ratio for the association of physical inactivity with risk of all-cause dementia in the first 10 years of follow-up in participant without dementia at baseline and from year 10 onwards in those without dementia at year 10 (age-, sex-, ethnicity and SES/education-adjusted random-effects meta-analysis)

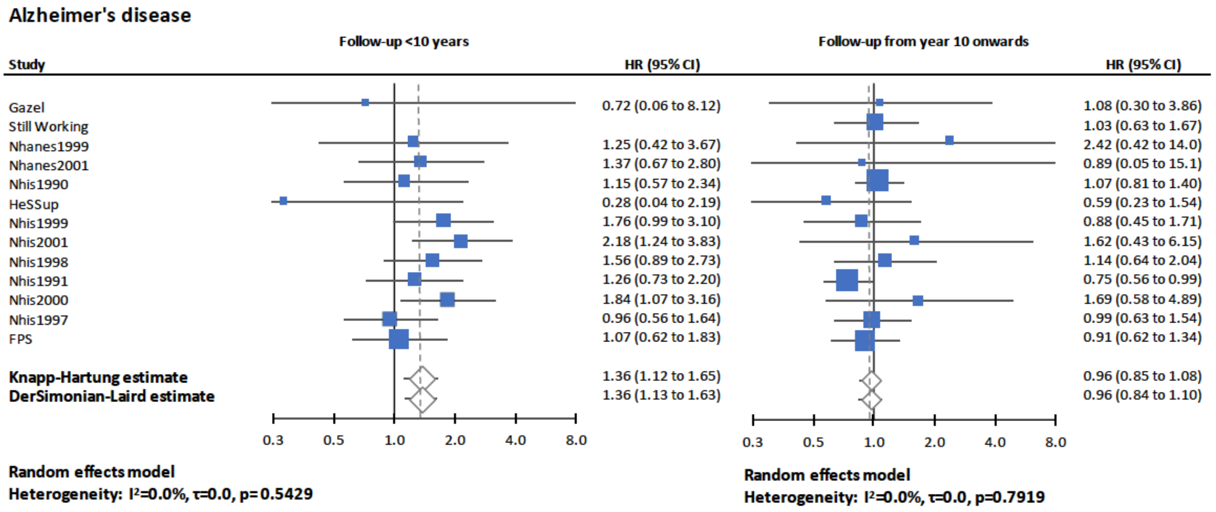

eFigure 3. Hazard ratio for the association of physical inactivity with risk of Alzheimer’s disease in the first 10 years of follow-up in participant without dementia at baseline and from year 10 onwards in those without dementia at year 10 (age-, sex-, ethnicity and SES/education-adjusted random-effects meta-analysis)

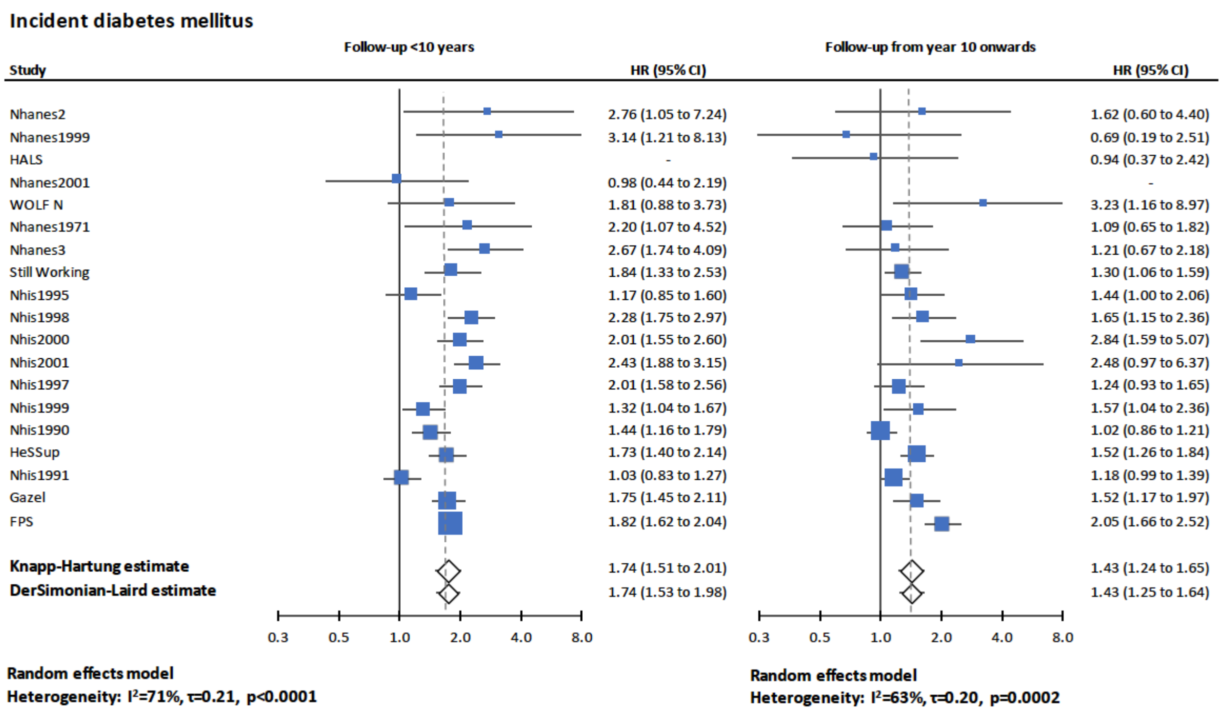

eFigure 4. Hazard ratio for the association of physical inactivity with risk of diabetes mellitus in the first 10 years of follow-up in participant without diabetes at baseline and from year 10 onwards in those without diabetes at year 10 (age-, sex-, ethnicity and SES/education-adjusted random-effects meta-analysis)

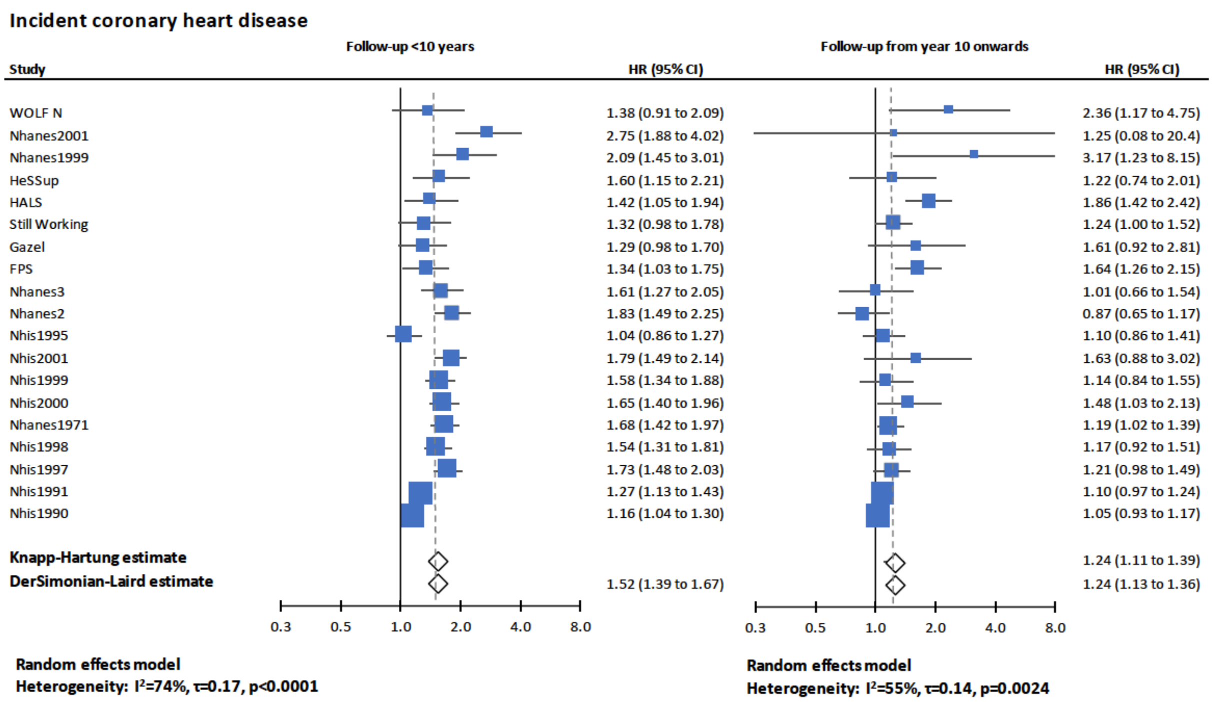

eFigure 5. Hazard ratio for the association of physical inactivity with risk of coronary heart disease in the first 10 years of follow-up in participant without the disease at baseline and from year 10 onwards in those without the disease at year 10 (age-, sex-, ethnicity and SES/education-adjusted random-effects meta-analysis)

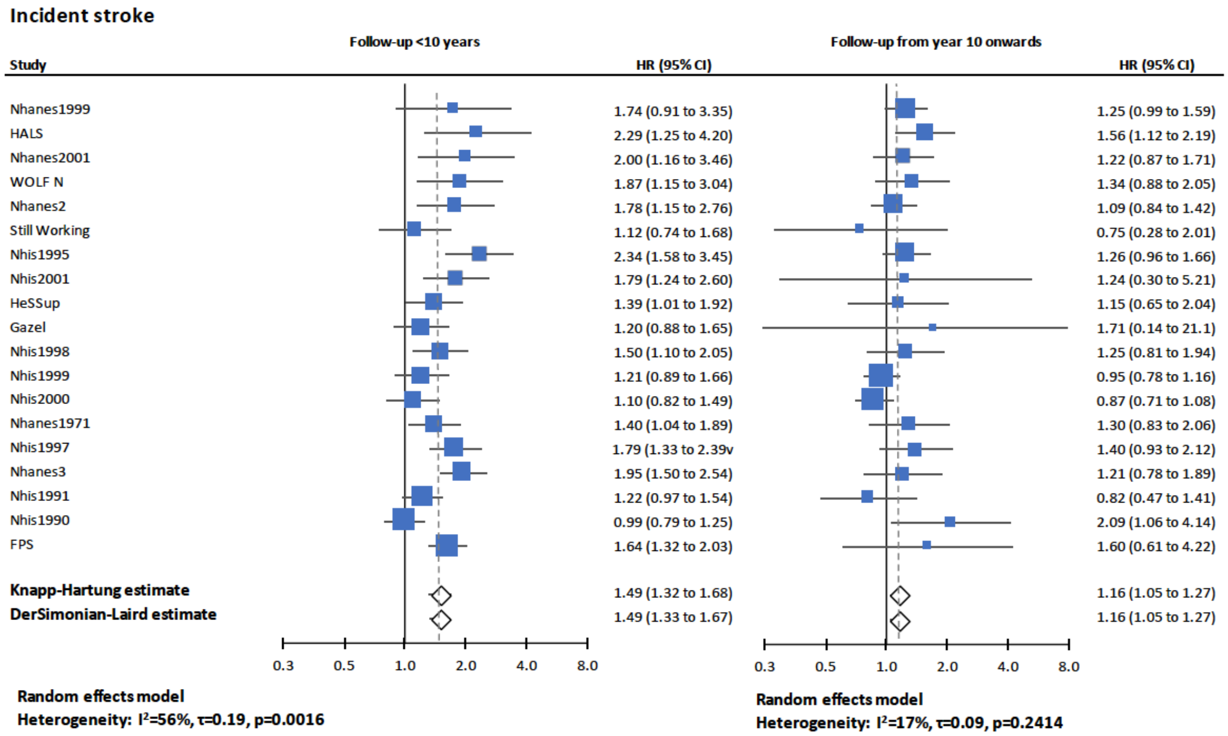

eFigure 6. Hazard ratio for the association of physical inactivity with risk of stroke in the first 10 years of follow-up in participant without stroke at baseline and from year 10 onwards in those without stroke at year 10 (age-, sex-, ethnicity and SES/education-adjusted random-effects meta-analysis)

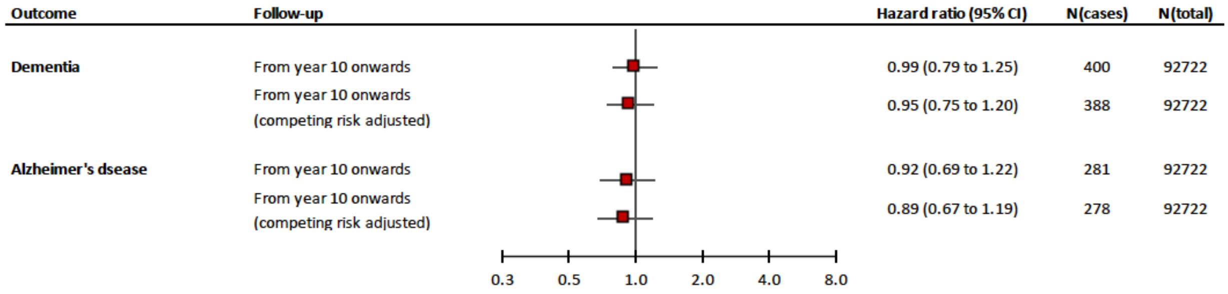

eFigure 7. Age-, sex-, ethnicity and SES/education-adjusted hazard ratio for the association of physical inactivity with risk of dementia and Alzheimer's disease from year 10 onwards in participants without dementia at year 10, before and after adjustment for competing risk of death

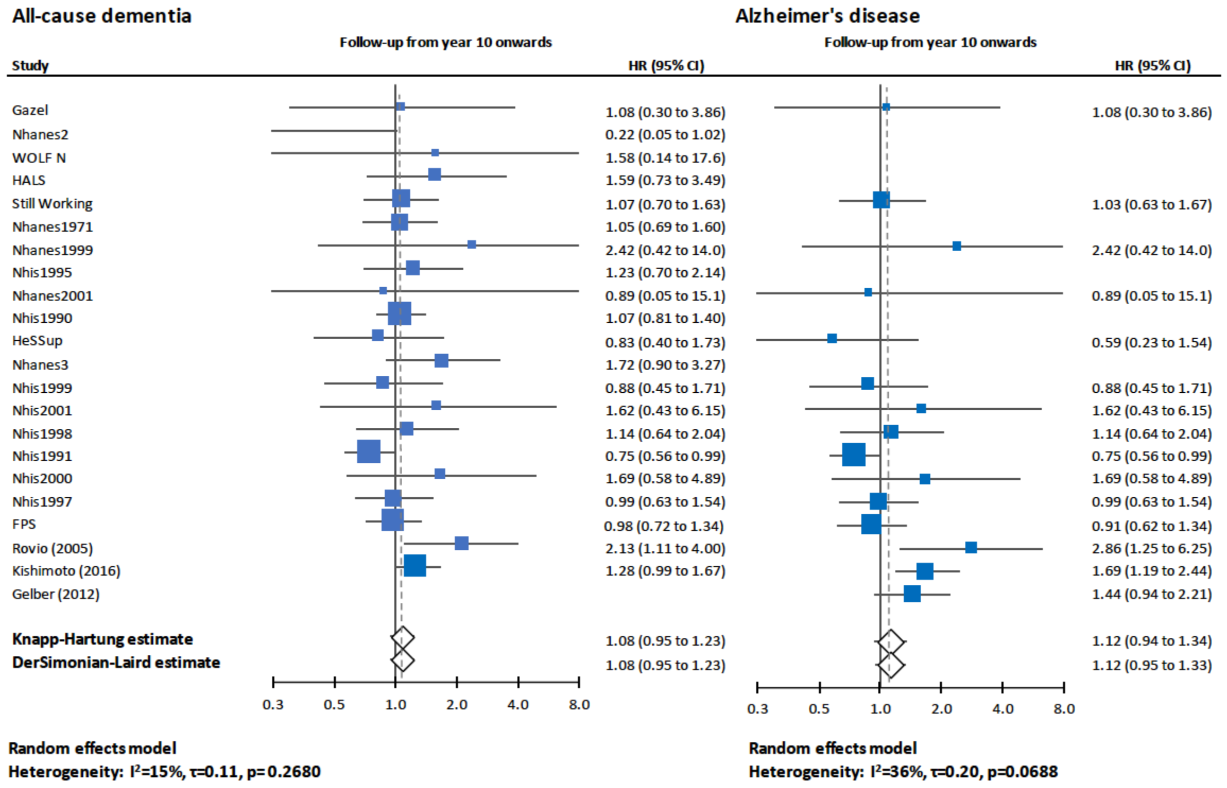

eFigure 8. Hazard ratio for the association of physical inactivity with risk of dementia and Alzheimer's disease from year 10 onwards in participants without dementia at year 10 in the present study and those for the association during a long follow-up in three previously published studies (random-effect meta-analysis)

References

1. VanderWeele TJ. Mediation Analysis: A Practitioner's Guide. *Annual review of public health* 2016; **37**: 17-32.
2. Lee IM, Shiroma EJ, Lobelo F, et al. Effect of physical inactivity on major non-communicable diseases worldwide: an analysis of burden of disease and life expectancy. *Lancet* 2012; **380**(9838): 219-29.
3. Sattelmair J, Pertman J, Ding EL, Kohl HW, 3rd, Haskell W, Lee IM. Dose response between physical activity and risk of coronary heart disease: a meta-analysis. *Circulation* 2011; **124**(7): 789-95.
4. Lear SA, Hu W, Rangarajan S, et al. The effect of physical activity on mortality and cardiovascular disease in 130 000 people from 17 high-income, middle-income, and low-income countries: the PURE study. *Lancet* 2017.
5. Chatterjee S, Peters SA, Woodward M, et al. Type 2 Diabetes as a Risk Factor for Dementia in Women Compared With Men: A Pooled Analysis of 2.3 Million People Comprising More Than 100,000 Cases of Dementia. *Diabetes care* 2016; **39**(2): 300-7.
6. Wolters FJ, Segufa RA, Darweesh SKL, et al. Coronary heart disease, heart failure, and the risk of dementia: A systematic review and meta-analysis. *Alzheimer's & dementia : the journal of the Alzheimer's Association* 2018; **14**(11): 1493-504.
7. Kuzma E, Lourida I, Moore SF, Levine DA, Ukoumunne OC, Llewellyn DJ. Stroke and dementia risk: A systematic review and meta-analysis. *Alzheimer's & dementia : the journal of the Alzheimer's Association* 2018; **14**(11): 1416-26.
8. Hartung J, Knapp G. A refined method for the meta-analysis of controlled clinical trials with binary outcome. *Statistics in medicine* 2001; **20**(24): 3875-89.
9. Cornell JE, Mulrow CD, Localio R, et al. Random-effects meta-analysis of inconsistent effects: a time for change. *Annals of internal medicine* 2014; **160**(4): 267-70.
10. Rucker G, Schwarzer G, Carpenter JR, Schumacher M. Undue reliance on I(2) in assessing heterogeneity may mislead. *BMC Med Res Methodol* 2008; **8**: 79.
11. Royston P, Parmar MK. Flexible parametric proportional-hazards and proportional-odds models for censored survival data, with application to prognostic modelling and estimation of treatment effects. *Stat Med* 2002; **21**(15): 2175-97.
12. Akaike H. Information theory and an extension of the maximum likelihood principle. In: Petrov BN, Csaki F, eds. Second International Symposium on Information Theory. Budabest; 1973: 267–81.
13. Frost C, Thompson SG. Correcting for regression dilution bias: comparison of methods for a single predictor variable. *Journal of Royal Statistical Society A* 2000; **163**(Part 2): 173-89.
14. Fine JP, Gray RJ. A Proportional Hazards Model for the Subdistribution of a Competing Risk. *Journal of the American Statistical Association* 1999; **94**: 496-509.
15. Allison PD. Survival Analysis Using the SAS System: A Practical Guide. Cary, NC: SAS Institute Inc, 1995.
16. Rovio S, Kareholt I, Helkala EL, et al. Leisure-time physical activity at midlife and the risk of dementia and Alzheimer's disease. *The Lancet Neurology* 2005; **4**(11): 705-11.
17. Kishimoto H, Ohara T, Hata J, et al. The long-term association between physical activity and risk of dementia in the community: the Hisayama Study. *European journal of epidemiology* 2016; **31**(3): 267-74.
18. Gelber RP, Petrovitch H, Masaki KH, et al. Lifestyle and the risk of dementia in Japanese-american men. *J Am Geriatr Soc* 2012; **60**(1): 118-23.
